# Effect of graded inclusion of black soldier fly (*Hermetia illucens,* Linnaeus, 1758) pre-pupae meal in diets for gilthead seabream (*Sparus aurata,* Linnaeus, 1758) on gut microbiome and liver morphology

**DOI:** 10.1007/s10695-025-01485-z

**Published:** 2025-04-22

**Authors:** Marco Basili, Basilio Randazzo, Letteria Caccamo, Stefano Guicciardi o Guizzardi, Martina Meola, Anna Perdichizzi, Grazia Marina Quero, Giulia Maricchiolo

**Affiliations:** 1 Department of Biological, Geological and Environmental Sciences (BIGEA), University of Bologna, Piazza di Porta S. Donato 1, 40126 Bologna, Italy; 2 IRBIM-Institute for Marine Biological Resources and Biotechnologies, CNR-National Research Council, Largo Fiera Della Pesca 1, 60125 Ancona, AN Italy; 3 IRBIM-Institute for Marine Biological Resources and Biotechnologies, CNR-National Research Council, Spianata S. Raineri 86, 98122 Messina, Italy; 4 National Biodiversity Future Center (NBFC), Piazza Marina 61, 90133 Palermo, Italy

**Keywords:** *Hermetia illucens*, Aquafeed, Gilthead seabream, Microbiota, Liver

## Abstract

Over the last decades, an insect meal has received great attention for finfish diets, due to its nutritional composition and low ecological footprint. In the present study, we assessed the response of gut microbiota composition and liver histology of gilthead seabream (*Sparus aurata*) fed four experimental diets including the black soldier fly (*Hermetia illucens*) meal (HI) used to replace 0 (HI0), 25 (HI25), 35 (HI35) and 50 (HI50) percent of fish meal in a 131-day feeding trial. At the end of the experiment, a remarkable change in gut microbiota composition related to HI inclusion was observed, with a preponderance of Cyanobacteriain the control and low HI groups (HI0, HI25) while Chloroflexi became prevalent in the higher HI inclusion groups (HI35, HI50). Predictive analysis on bacterial metabolic pathways showed a clear separation between HI0–HI25 and HI35–HI50 groups. The microbiota shifts observed suggest a pivotal role of HI in inducing a bacterial-mediated physiological response in this fish species, probably due to chitin content and the fatty acid profile of this ingredient. Liver histology showed a higher hepatocyte size in fish from the HI50 group, suggesting lipid dysmetabolism due to the HI meal fatty acid profile, while a marginal adaptive response was observed in the HI25 group. In conclusion, while up to 25% inclusion of black soldier fly meal showed limited adverse effects, 50% HI dietary inclusion is not recommended in gilthead seabream diet, since possible alteration in lipid deposition, particularly at hepatic level, were highlighted in this fish species.

**Supplementary Information:**

The online version contains supplementary material available at 10.1007/s10695-025-01485-z.

## Introduction

Currently, aquaculture is the fastest growing animal production sector worldwide, and it is considered one of the more sustainable practices to produce animal proteins for human consumption (Hilborn et al. 2018; Poore and Nemecek 2018). However, the exponential increase in food demand, combined with the need to reduce the ecological footprint of aquaculture practices, is driving the need to find sustainable alternatives to traditional ingredients, such as fish meal (FM) in aquafeed formulations (FAO 2022; Tacon et al. 2022; UN DESA 2023; Naylor et al. 2023). The production of aquafeed, intended for commercial aquaculture, is strictly dependent from high-protein ingredients supply, which represents one of the main bottlenecks for the further development of the sector (Hua et al. 2019).

Traditionally, FM was considered the optimal protein source for fish diets due to its well-balanced amino acid and fatty acid (FA) profile, matching nutrient’s requirements in carnivorous fish species (Tacon and Metian 2015). However, considering the depletion of natural fish stocks, and the high costs (Olsen and Hasan 2012; Jannathulla et al. 2019), FM has been gradually replaced with other readily available and cost-effective protein sources, such as plant-derived ones, including soybean, rapeseed meal, moong, guar and sorghum meals (Watanabe et al. 1993; Gatlin et al. 2007; Hua et al. 2019; Macusi et al. 2023). Despite undeniable advantages, high levels of vegetable ingredients are tolerated by a limited number of cultured fish species, particularly herbivorous and omnivorous ones (Liu et al. 2021, Howlader et al. 2023). On the contrary, in carnivorous species, a tolerability threshold for dietary plant–derived ingredients is low since they are held responsible of negative side effects on fish gut health, due to the substantial content in non-digestible carbohydrates, anti-nutritional factors and unbalanced amino acid profile (Gatlin et al. 2007; Collins et al. 2013; Pahlow et al. 2015). Moreover, not least, some plant-derived ingredients used for aquafeed production are in direct competition with human consumption (Qin et al 2022). As a consequence, in the last decades, finding novel ingredients for aquafeeds has become mandatory (Gasco et al. 2018; Nugroho and Nur 2018; Galkanda-Arachchige et al. 2020; Aragão et al. 2022; Carvalho et al. 2023). Remarkable progresses have been moved toward the use of novel protein sources, including livestock’s by-products (poultry meal, blood meal, and hydrolyzed feather meal) and low-trophic organisms, such as yeasts, micro and macro algae and invertebrates (Albrektsen et al. 2022; Zarantoniello et al. 2022a; Randazzo et al. 2022).

In addition to plant protein sources, after the authorization by the EC Regulation No. 893/2017, insects are seen as promising alternatives, thanks to their high protein content and environmental benefits (Maiolo et al. 2020; van Zanten et al. 2014; Maulu et al. 2022). Among insects, black soldier fly (*Hermetia illucens*, HI) stood out for its richness in protein (60–70% on a dry matter basis (DM)), essential amino acid (EAA) profile, as well as bioactive compounds, including vitamins (especially B12), minerals (iron and zinc) medium-chain fatty acids (mainly Lauric acid, C12), chitin and antimicrobial peptides, which have been proven to improve fish health by modulating gut microbiome in different fish species (Barroso et al. 2014; Henry et al. 2015; Benhabiles et al. 2012; Komi et al. 2018; Antonopoulou et al. 2019; Rimoldi et al. 2019; Stenberg et al. 2019; Terova et al. 2019, 2021; Basto et al. 2020; Oteri et al. 2022; Hoseinifar et al. 2024). Particularly, lauric acid is known to exert and inhibitory activity against Gram-positive such as *Clostridium perfringens*, *Staphylococcus aureus* and D-Streptococci (Skrivanova et al. 2006; Spranghers et al. 2018), thus reducing potentially pathogenic Gammaproteobacteria (Borrelli et al. 2021; Rimoldi et al. 2024). Also, chitin, and its oligomers, as the main component of insect’s exoskeleton, acts as insoluble dietary fiber, resembling cellulose structure, and has been shown to possess probiotics properties (Rangel et al. 2022; Weththasinghe et al. 2022; Rimoldi et al. 2023) and bacteriostatic properties against several harmful Gram-negative bacteria (Qin et al. 2014; Nawaz et al. 2018). Although different effects of dietary *H. illucens* meal on fish gut microbiome were reported, and results are still not conclusive (Hossain et al. 2023), improved bacterial richness and abundance were often recorded, particularly of beneficial lactic acid bacteria (Huyben et al. 2019; Weththasinghe et al. 2022; Biasato et al. 2022; Hasan et al. 2023).

In the last years, *H. illucens* has been tested on several fish species, including experimental models, such as zebrafish (*Danio rerio*), and the most widely farmed fish species worldwide, such as Atlantic salmon (*Salmo salar*), rainbow trout (*Oncorhynchus mykiss*), mirror carp (*Cyprinus carpio*), hybrid tilapia (Oreocromis niloticus × O. mozambique), African catfish (*Clarias gariepinus*), European seabass (*Dicentrarcus labrax*), Japanese seabass (*Lates calcarifer*), Siberian sturgeon (*Acipenser baerii*), meagre (*Argyrosomus regius*) and gilthead seabream (Zarantoniello et al. 2018; Abdel-Tawwab et al. 2020; Adeoye et al. 2020; Bruni et al. 2020a; Bruni et al. 2020b; Fawole et al. 2020; Fisher et al. 2020; Li et al. 2020; Randazzo et al. 2020a; Ratika et al. 2020; Xu et al. 2020; Abdel-Latif et al. 2021; Abu Bakar et al. 2021; Agbohessou et al. 2021; Caimi et al. 2021; Guerreiro et al. 2021; Hender et al. 2021; Hoc et al. 2021; Moutinho et al. 2021; Randazzo et al. 2021; Rimoldi et al. 2021; Tippayadara et al. 2021; Zarantoniello et al. 2021; Di Rosa et al. 2023; Oteri et al. 2022; Randazzo et al. 2022, Rangel et al. 2022; Gai et al. 2023; Busti et al. 2024; Randazzo et al. 2023; Rimoldi et al. 2024). On the other hand, the use of *H. illucens* meal in aquafeeds still presents some limitation due to its lipid content (up to 50% on DM) and profile, with a predominance of short chain-saturated fatty acids (SC-SFAs) at expense of essential n-3 long-chain polyunsaturated fatty acids (LC-PUFA) (Barragan-Fonseca et al. 2017; Magalhães et al. 2017; Hoc et al. 2020). While many freshwater species are able to convert shorter chain precursors in highly unsaturated FAs through the activation of specific elongase and desaturase enzymatic patterns (Glencross 2009; Tocher 2010), marine species such as turbot, sea bass and seabream are assumed to have a weak capacity to activate SC-SFAs conversions, thus requiring the preformed HUFAs in their diet (Turkmen et al. 2019; Ferosekhan et al. 2020; Izquierdo et al. 2008; Li et al. 2014; Vagner and Santigosa 2011). These subtle biochemical differences between fish species can be at the base of systemic physiological impairments, particularly affecting organs involved in lipids storage and metabolism (Vargas-Abúndez et al. 2019). As a consequence, some fish species may suffer high levels of *H. illucens* meal inclusion in the diet by exhibiting a large set of physiological responses. To date, most of the studies have been made on salmonids (Terova et al. 2019, 2021; Rimoldi et al. 2019, 2021; Biasato et al. 2022), while only recently gut microbiome changes induced by *H. illucens* meal dietary inclusion in gilthead seabream (*Sparus aurata*) have been explored (Panteli et al. 2021; Rangel et al. 2022; Busti et al. 2024; Rimoldi et al. 2024). On the contrary, most of the studies performed so far on gilthead seabream were focused on filet quality, enzymatic activities, serum parameters, gut health and liver condition (Fabrikov et al. 2021; Di Rosa et al. 2023; Karapanagiotidis et al. 2023; Gai et al. 2023; Randazzo et al. 2021, 2023). The liver, in particular, has been often considered as a target marker organ of nutritional conditions when testing new diets in fin fish species, playing a pivotal role in most of the fish metabolic pathways (Bakke et al. 2010; Bruslé et al. 2017; Bruni et al. 2020b; Vargas-Abúndez et al. 2019). Histology represents one of the golden standards to analyze liver condition, and different approaches are adopted to evaluate morphological changes in this organ in fish, all considering hepatocytes lipid deposition and parenchymal structure changes, including circulatory congestion and inflammatory influx (Bernet et al. 1999; Zarantoniello et al. 2022b; Donadelli et al. 2024). However, knowledge on the interactions between insect-based diets, gut microbiome and liver condition is still fragmentary in fish. Gilthead seabream is one of the most important cultured fish species for the European aquaculture market, representing more than 16% of total production in terms of volume and 26% in terms of value (FEAP 2023).

While many studies have focused on the effect of HI meal on fillet quality, enzyme parameters and gut health, in the present study the effect of increasing dietary level of *H. illucens* meal (replacing up to 50% FM) was assessed on gilthead seabream (*Sparus aurata* Linnaeus, 1758) gut microbiome and liver histology.

## Material and methods

### Ethics

Feeding trial details are reported in a previous study (Di Rosa et al. 2023). The feeding trial experiment and all the procedures involving animals were carried out in strict accordance with Italian legislation (D.Lgs. n.26/2014) implementing the European Directive 2010/63/EU (European Directive 2010, 2014) on the protection of animals used for scientific purposes. The experimental protocol was authorized by the Italian Ministry of Health (n. 491/2019-PR of the 9 July 2019).

### Experimental diets

The experimental diets used in the feeding trial are reported in a previous study (Di Rosa et al. 2023). Briefly, four isoproteic (42.7%), isolipidic (approximately 18.6%) and isoenergetic (approximately 22 MJ kg^−1^), and diets were formulated based on the nutritional requirements of *Sparus aurata* (NCR 2012). A FM-based diet (HI0) was used as control (FM 250 g kg^−1^). Other three diets (HI25, HI35 and HI50) were formulated to replace 25%, 35% and 50% FM with defatted *Hermetia illucens* meal (79, 110 and 157 g kg^−1^, respectively in the diets). Where necessary, the diets were supplemented with essential amino acids (L-lysine, L-tryptophan, DL-methionine and L-taurine) to meet seabream nutrient requirements (National Research Council (NRC) 2012). Diets were produced at SPAROS Lda (Olhao, Portugal) by extrusion and a pellet size of 4 mm was obtained, as previously described (Di Rosa et al. 2023). The ingredients and the nutritional profile of the diets are shown in Table 1. For the chemical composition, the nitrogen-free extract and the gross energy (GE, MJ/kg) content, please refer to the work from Oteri and colleagues (Oteri et al. 2021).

**Table 1:** Diet ingredients and proximate composition of the experimental diets as reported in Di Rosa et al. (2023)

	HI0	HI25	HI35	HI50
Ingredients, % as fed				
Fish meal	25.00	18.75	16.25	12.50
* Hermetia illucens* meal	0	7.90	11.00	15.70
Soy protein concentrate	5.00	5.00	5.00	5.00
Wheat gluten	5.00	5.00	5.00	5.00
Corn gluten	5.00	5.00	5.00	5.00
Soybean meal 48	15.00	15.00	15.00	15.00
Rapeseed meal	5.00	5.00	5.00	5.00
Wheat meal	17.45	15.17	14.21	12.88
Whole peas	4.00	4.00	4.00	4.00
Fish oil	5.00	5.00	5.00	5.00
Rapeseed oil	10.00	9.80	9.80	9.80
Vitamin and mineral premix	1.00	1.00	1.00	1.00
Vitamin C35	0.03	0.03	0.03	0.03
Vitamin E50	0.02	0.02	0.02	0.02
Antioxidant	0.30	0.30	0.30	0.30
Sodium propionate	0.10	0.10	0.10	0.10
MCP, monocalcium phosphate	1.50	2.20	2.50	2.80
L-Lysine	0.30	0.35	0.37	0.40
L-Tryptophan	-	0.03	0.04	0.05
DL-Methionine	0.10	0.15	0.18	0.22
L-Taurine	0.20	0.20	0.20	0.20
Proximate analysis, g/100 g as fed				
Dry matter	92.33	92.78	92.90	92.64
Crude protein	42.7	42.7	42.7	42.7
Crude fat	18.6	18.6	18.6	18.7
Crude fiber	2.3	2.2	2.2	2.1
Ash	9.3	9.3	9.4	9.3
NFE*	19.43	19.98	20.00	19.84
Gross energy, mj kg^−1^	22.0	21.9	21.9	21.9

*Nitrogen-free extract, nfe (%) = 100—(%crude protein + %crude fat + %crude fiber + %ash)

Determined by calorimetric bomb

### Fish and feeding trial

Feeding trial details are reported in a previous study (Di Rosa et al. 2023). Briefly, *Sparus aurata* specimens (*n* = 312), obtained by the Ittica Caldoli Company (Lesina, Foggia, Italy), were transferred to the IRBIM-CNR facility (Messina, Italy) and housed in a 4.5-m^3^ tank connected to an open recirculating system equipped with a sand mechanical filter and UV lamp. Chemico-physical parameters were daily monitored (pH, 7.8 ± 0.5; dissolved oxygen, 7.8 ± 0.5 mg L^−1^; temperature, 20.3 ± 8.4 °C; TAN < 0.02 mg L^−1^; N-NO2 < 1.0 mg L^−1^). Photoperiod followed local seasonal changes (February–August; latitude: 38° 110390′ 48 N). Fish were initially fed a commercial diet (46% protein, 16% fat, 20.7% lipids, 2.3% crude fiber; Aller Blue Omega 3 mm; Aller Aqua Company, Christiansfeld, Denmark) for 1 month. After acclimation, fish were sedated (MS222, Tricaine Pharmaq; 25 mg L^′1^), individually weighted (average initial weight: 143.65 ± 25.94 g) and randomly divided into 12 fiberglass tanks of 1.4 m^3^ (*n* = 26 per tank) and assigned to the four experimental groups (HI0, HI25, HI35 and HI50) in triplicate. Fish were fed the experimental diets (0.8 to 1.5% body weight, according to the temperature and rationing table for this species), 6 days a week in two daily meals, for 131 days. Fish were weighed in bulk every 20 days to estimate fed provision during the experiment. At the end of the trial (T1), after a 24-h fasting period, all fish were sacrificed through an overdose of anesthetic (MS222, Tricaine Pharmaq; 0.5 g L^−1^) for gut and liver sampling.

### Gut microbiome sampling

For microbiome analysis, we collected *n* = 5 fish individuals at the beginning (T0) and *n* = 12 fish individuals at the end of the trial (T1); per each treatment, *n* = 3 individuals were collected. Samples from T0 were used in order to provide a picture of gut microbiome composition before feeding the test diets. For each fish, fore- and hindgut were separately collected. For each section, intestinal tissues, contents and mucosae were isolated by using sterile tools, including sterile scissors and scalpels. Intestinal contents (feces or digesta) were collected by gentle squeezing, while mucosae were collected by scraping. Tissue samples were rinsed with a sterile phosphate buffer solution to remove possible loosely attached microorganisms; samples were then immediately placed in sterile tubes. All types of samples were subsequently stored at − 20 °C until analyses.

### DNA extraction and sequencing

DNA from seabream feces, mucosae and tissues was extracted using the DNeasy PowerSoil Kit (Qiagen) as previously described in Quero et al. (2023). Extracted DNA samples were quantified using the NanoDrop ND-1000 (NanoDrop Technologies) spectrophotometer and subsequently stored at − 20 °C until processing. Gut microbiota composition was assessed through high-throughput sequencing of the 16S rRNA gene. In more detail, the V3–V4 hypervariable region of the 16S rRNA gene was amplified using the 341F-785R primer pair (Klindworth et al. 2013); the obtained PCR products were purified as described in Palladino et al. (2021). Nextera library indexing and preparation and Illumina MiSeq sequencing (2 × 300 bp paired-end protocol) were performed as previously described in Palladino et al. (2021). All the obtained raw sequences are submitted to the SRA—Sequence Read Archive (BioProject PRJNA1136692 Biosamples SAMN42447806-SAMN42447954).

### Sequencing data analysis

All primer and adapter sequences were removed from raw reads with the Cutadapt algorithm (Martin 2011). Subsequently, paired-end reads were imported in RStudio (RStudio Team 2020) and analyzed in the same environment using the DADA2 package (Callahan et al. 2016). Reads were quality checked and trimmed following the package instructions (at 220 and 200 bp for forward and reverse reads, respectively; max estimated error > 2 and 2 per 100 bp for forward and reverse reads, respectively). Paired-end reads were then merged in ASVs (amplicon sequence variants). We then identified and removed chimeric sequences from the dataset. Prokaryotic taxonomy was assigned using a native implementation of the naive Bayesian classifier method against the SILVA database (v138; https://www.arb-silva.de/documentation/release-138/). Chloroplast and eukaryotic sequences were then removed from the obtained ASV table.

### Liver histology

At the end of the trial, the liver was isolated on a subsample of 24 fish (two fish per tank, six fish per diet) and fixed in Bouin’s solution (Sigma-Aldrich, Italy) for 24 h. Afterward, samples were washed in 70% ethanol, dehydrated in graded ethanol solutions, clarified with xylene and embedded in solid paraffin. Sections of 5 µm thickness (three from each sample at 100-µm intervals) were obtained using a microtome and stained with haematoxylin & eosin (H&E). General histomorphology was first evaluated for the description of the hepatic tissue in all the specimens from the different experimental groups. Pictures from random-chosen fields for each stained section were photographed at 40 × magnification by means of a Leica DFC295 color camera and used for the hepatocytes and hepatocytes delocalized nuclei (peripheral nuclei) quantification. For each area acquired (75,840 µm^2^), counts were performed by the Images Analyzer software (Leica Las V4.9). Liver condition evaluation was performed live by two independent observers in a double-blinded examination, and a 0–3 score was assigned to each liver condition index considered, according to Donadelli et al. (2024), as modified by Bernet et al. (1999). The histological alterations considered for the assignment of the score to each index are reported in Table 2. Results were reported as the mean of the observation and analyzed as reported in the “Statistics” section.

**Table 2:** Histological alterations considered for the assignment of the liver condition indexes score

Liver condition index	Histological alteration
Circulatory disturbance	Sinusoid congestion
	Blood vessel congestion
	Hemorrhages
Regressive changes	Mild lipid accumulation
	Severe lipid accumulation
	Pycnotic nuclei
	Cord loss
	Vacuolar tissue degeneration
Progressive changes	Tissue necrosis
	Hepatocytes hyperplasia
	Hepatocytes hypertrophy
	Bile duct hypertrophy
Granulocytes infiltration	Scattered cells
	Tightly aggregated cells

### Statistics

For microbiome data, all statistical analyses were performed in Rstudio (RStudio Team 2020). The ASV table was normalized using the median value of the dataset with the *vegan* and *phyloseq* packages (Oksanen et al. 2013, McMurdie and Holmes 2013). For the analysis of alpha diversity, ASV richness was calculated. The occurrence of statistical differences among richness values in the different types of samples was assessed with ANOVA test (*stats* package) considering all possible comparisons. Non-metric multidimensional scaling (nMDS) was performed using a Jaccard dissimilarity matrix and average linkage approach and plotted with the *ggplot2* package (Wickham 2016). Significant differences in prokaryotic community composition among treatments and among fish tissues (i.e., anterior–posterior, fecal-mucosa-tissue) were calculated by using PERMANOVA through the *adonis* function (*vegan* package) in R, based on a Jaccard distance matrix calculated on the relative abundant ASV table. From this step, we excluded the starting time point samples, due to the higher diversity occurring in terms of richness and composition of the microbial community; this allowed us to focus our comparison specifically on the final time point and the resultant alterations in microbial communities induced by the different diets. A “Linear Discriminant Analysis Effect Size (LEfSe)” to find biomarkers of each group (i.e., CTRL-HI25 and HI35–HI50) was performed and plotted using the *ggplot2* package. For this analysis, all types of intestinal samples were considered together. The bioinformatic software PICRUSt (Phylogenetic Investigation of Communities by Reconstruction of Unobserved StatesPredicted Metagenome Analysis and Metabolic Reconstruction) (Langille et al. 2013) was used to perform metagenome prediction in each seabream microbiome sample, following the standard procedures (http://picrust.github.io/picrust/tutorials/genome_prediction.html#genome-prediction-tutorial) and using default settings. Briefly, PICRUSt uses a normalized ASV table of 16S rRNA gene data to predict metagenome functional content; this prediction is performed basing on pre-calculations for genes in the KEGG (Kyoto Encyclopedia of Genes and Genomes) database. Metagenomes were predicted from the normalized dataset (*predict_metagenomes.py* script) against the KEGG Orthology (KO) database. The contribution of various taxa to different KOs was computed with the script *metagenome_contributions.py*. Since some KEGG orthologs (KOs) can be represented in multiple pathways, we used the *categorize_by_function.py* script to collapse the predictions at the individual pathways level. Subsequently, we looked for those genes differentially abundant in the microbiome of seabream treated with the different diets. We used the *ggpicrust2* R package (Yang et al. 2023) for the statistical analysis and the visualization of acquired results.

For liver histology analyses, ANOVA analysis with pairwise post hoc comparison was used to assess the impact of the “Diet” factor on the parameter values. Before the test, the homogeneity of variance was examined using Levene’s test (Lomex and Hahs-Vaughn 2012) In case of homoscedasticity, the standard parametric ANOVA was applied with Tukey HSD post hoc comparisons, while in case of heteroscedasticity, a nonparametric ANOVA with games-howell post hoc comparisons (Maxwell et al. 2018) was employed. For the latter scenario, we used the *oneway* function of the R package *userfriendlyscience* 0.7.2 (Peters 2018) All statistical analyses were carried out using the freely available statistical software R version 4.3.3. In all tests, a *p*-value equal to or less than 0.05 was considered statistically significant.

## Results

### Microbiota

Out of a total of 102 samples collected from fish individuals, 97 yielded DNA of sufficient quality and quantity for 16S rRNA gene sequencing. Of these, 26 (*n* = 2 anterior content, *n* = 5 anterior tissue, *n* = 4 anterior mucosae, *n* = 5 posterior content, *n* = 5 posterior tissue, *n* = 5 posterior mucosae) DNA samples were collected from fish individuals at T0 and 71 from fish individuals collected from the different treatments at T1 (*n* = 12 anterior content, *n* = 12 anterior tissue, *n* = 12 anterior mucosae, *n* = 11 posterior content, *n* = 11 posterior tissue, *n* = 12 posterior mucosae).

**Fig. 1:**
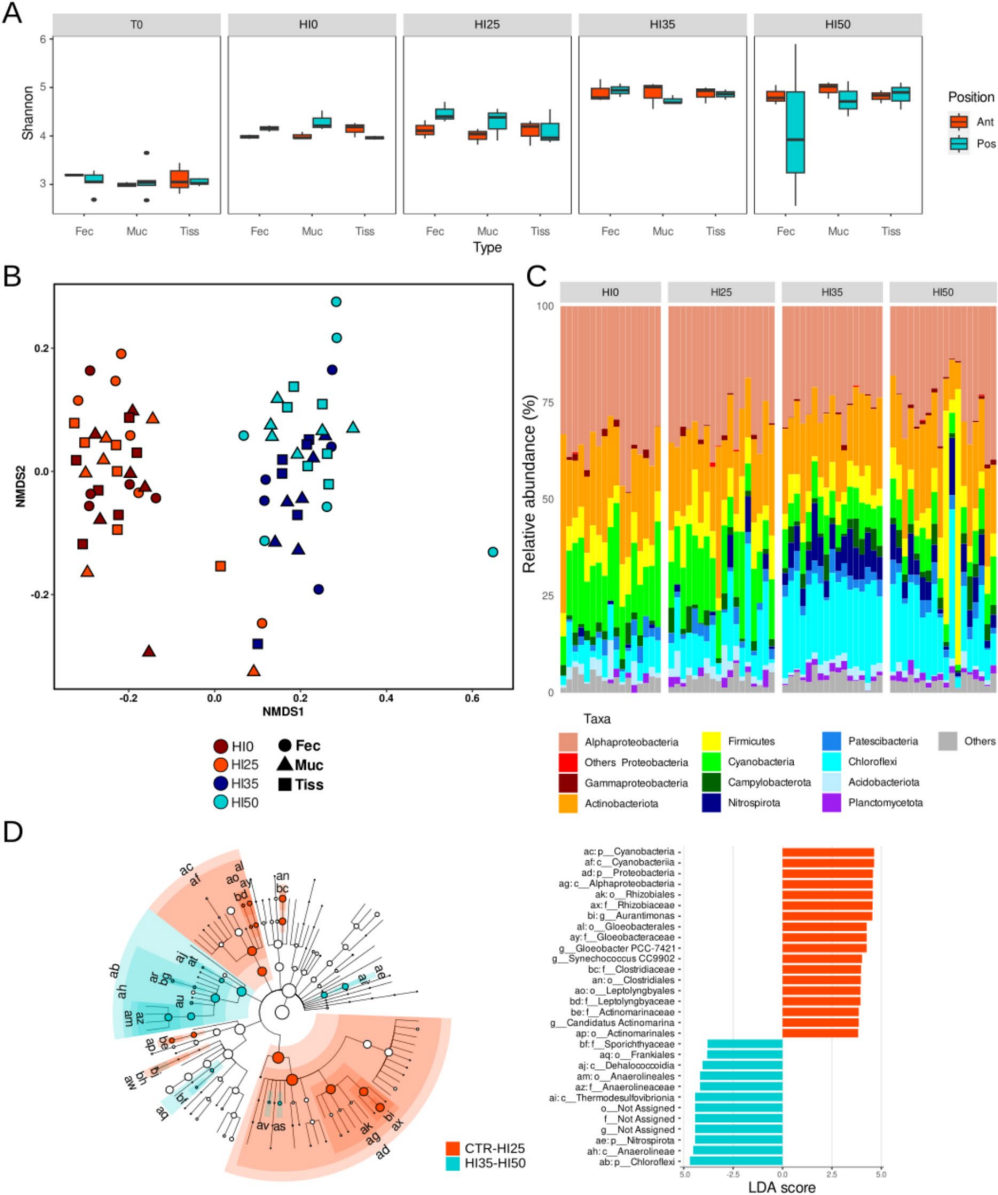
Panel **A** Boxplot reporting Shannon index values calculated for the different types of samples, treatment and sampling points (Fec, intestinal content, i.e., feces or digesta; Muc, mucosa; Tiss, intestinal tissues). Panel **B** Non-metric multidimensional scaling (NMDS) ordination of fish microbiome community composition based on Jaccard dissimilarity matrix. Colors indicate diet and shapes indicate the type of gut tissue. Panel **C** Microbiome community composition in the different fish tissues and treatments at the phylum and class level (for Proteobacteria only). Data are reported as relative abundance (%). Taxa with an average relative abundance across all samples < 1% were aggregated as “Others”. Left panel **D** Taxonomic cladogram plotted from LEfSe analysis showing differentially abundant taxa (*p* < 0.01, LDA > 3.0) in gut microbiomes from HI0–HI25 to HI35–HI50 groups. Significantly discriminant taxa nodes are colored according to the group; a two-letter code per each significant node corresponds to the taxonomic identification reported in the *y*-axis of Right panel **D** Branch areas are shaded according to the highest ranked group for that taxon. Not significantly discriminant taxa are represented in white. Right panel: LDA scores (log10) for the top 30 taxa in gut microbiome samples from HI0–HI25 to HI35–HI50 groups

The analysis of alpha diversity, as measured by the Shannon index, revealed a significant difference between samples at the beginning (T0) and at the end (T1, including HI0, HI25, HI35 and HI50) of the feeding trial (ANOVA *p* < 0.001), with lower diversity values observed at T0 (Fig. 1A). Within the samples collected at T1, samples from HI0 and HI25 exhibited lower alpha-diversity values compared to those from HI35 and HI50 groups (Fig. 1A). No significant differences were observed between fore- and hindgut tissue microbiomes from the same time point, diet and type of sample (i.e. tissue, content, mucosa).

At the community composition level, non-metric multidimensional scaling (NMDS) (Fig. 1B) underlined a clear separation into two main groups, one including microbiome samples from HI0 and HI25, and the second including those from HI35 and HI50 groups (adonis, *p* < 0.001). Within each group, no further separation between treatments was observed, i.e. HI0 and HI25 showed a similar microbiome community composition, as well as HI35 and HI50 (Fig. 1B).

Proteobacteria (avg. 31.0 ± 7.4%) and Actinobacteriota (avg. 19.9 ± 5.9%) dominated fish microbiomes at all time points and in all treatments (Fig. 1C). Within Proteobacteria, we observed high contributions of Alphaproteobacteria (avg. 30.5 ± 7.3%), which showed a slight decrease, although not significant, with increasing HI percentages in the tested feedings. On the other side, relative abundances of Cyanobacteria showed marked differences between HI0–HI25 (avg. 16.1 ± 5.8%) and HI35–HI50 (avg. 7.5 ± 2.4%) groups, with higher contributions in low HI treatments than in the latter. HI35 samples showed the highest abundances of Chloroflexi (avg. 20.4 ± 2.8%) compared to HI0 (avg. 6.1 ± 3.0%), HI25 (avg. 10.9 ± 7.7%) and HI50 (avg. 16.5 ± 8.5%), and represented the main discriminant taxon between HI35 and all the other treatments. Among other most abundant phyla, Nitrospirota also showed higher abundances in HI35 and HI50 communities (avg. 6.1 ± 3.3%) than in HI0 and HI25 (avg. 0.8 ± 2.17%) (Fig. 1C). Within treatments, no differences between different gut tissues (i.e. fore- and hindgut, or intestinal contents, mucosa and tissue) were highlighted. Firmicutes statistically decreased from T0 (data not shown, avg. 66.4 ± 13.1%) to T1. However, in T1 samples, after removing posterior content 10 and 12, which showed the highest relative abundances of Firmicutes (more than 50% of the total community) and were therefore identified as outliers, we found that Firmicutes were statistically higher in HI0 and HI25 communities than in HI35 and HI50 (ANOVA *p* < 0.001).

In order to identify microbial biomarkers that significantly characterized the different treatments, we applied the LEfSe (linear discriminant analysis effect size) algorithm (Segata et al. 2018). To this aim, we compared fish gut microbiomes from the two groups of samples, HI0–HI25 to HI35–HI50 (Fig. 1D), identified by the NMDS analysis. Proteobacteria (Alphaproteobacteria), Cyanobacteria and Chloroflexi were identified as those phyla exhibiting differential abundances in gut samples from the two groups. Within Alphaproteobacteria, the genus *Aurantimonas* (fam. Rhizobiaceae) was statistically enriched in the HI0-HI25 group (avg., 12.43 ± 3.6%). Anaerolineaceae (average 4.0 ± 3.4%) were identified as those members of Chloroflexi characterizing HI35–HI50 samples (Fig. 1D). When considering separately the four treatments, LEfSe identified several taxa within Alphaproteobacteria as differentially abundant in HI0, Cyanobacteria in HI25 and Chloroflexi in HI35 samples (Figure S1).

**Fig. 2:**
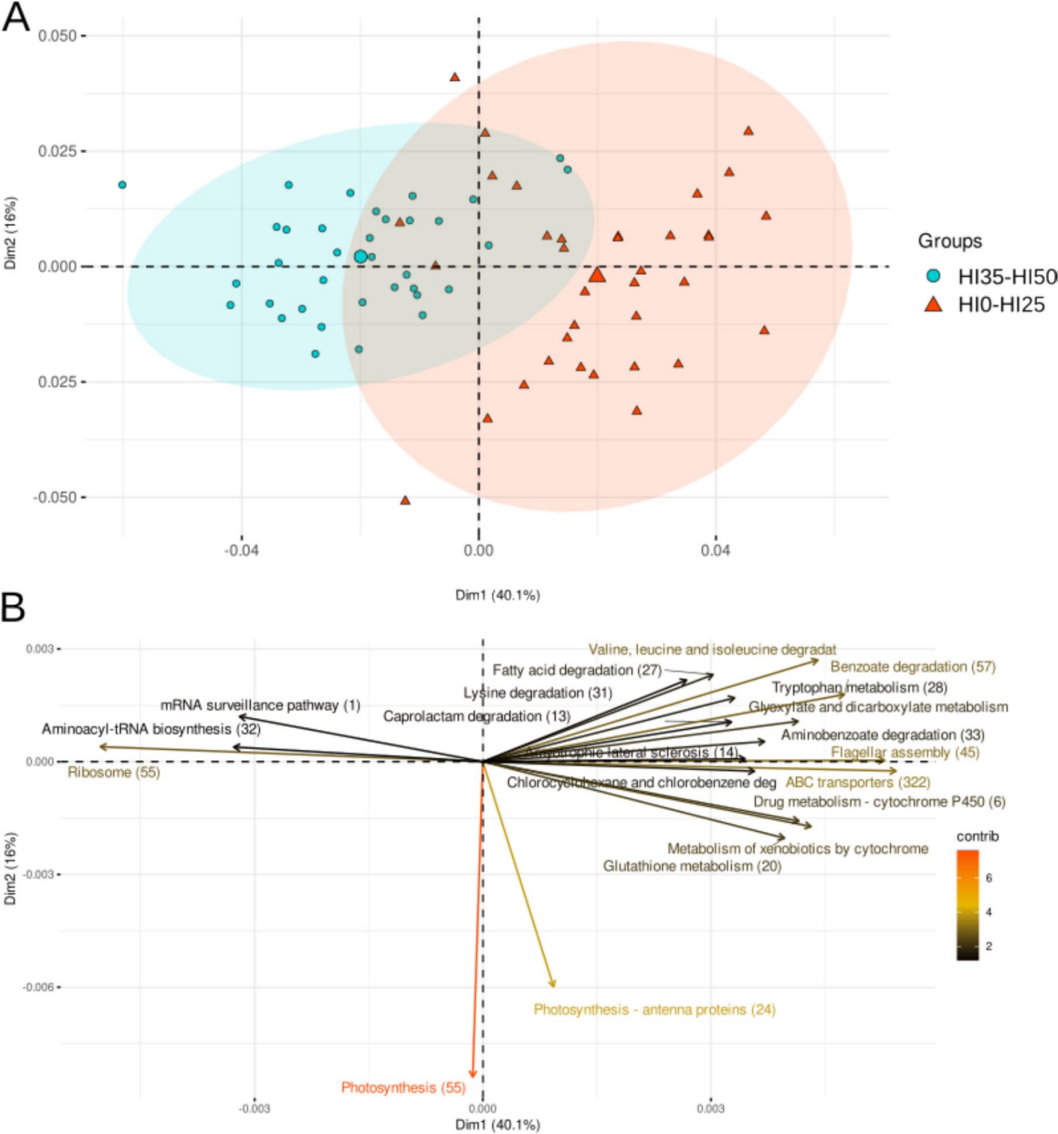
Top 20 discriminant KEGG orthology (KO) genes from the PICRUSt analysis, colored by contribution. The *x*-coordinate represents the first principal component, the *y*-coordinate represents the second principal component, and the percentage represents the contribution of the principal component to the sample variance

To address the potential functioning of the gut microbiomes associated with the different treatments, PICRUSt-predicted metagenomes of the HI0–HI25 and HI35–HI50 groups were analyzed (Fig. 2A). The principal component analysis (PCA) biplot, constructed using predicted gene abundances, distinctly delineated the gut microbiome profiles of individuals within the HI0–HI25 and HI35–HI50 groups along the *x*-axes of the representation. Notably, the separation was driven by differential gene abundances associated with genetic translation processes, including Ribosome, mRNA surveillance, and aminoacyl-tRNA biosynthesis, predominantly observed in the HI35–HI50 microbiomes. Conversely, genes related to flagellar assembly and membrane transport discriminate the sample distribution, constituted the predominant gene families in the HI0–HI25 microbiomes, accompanied by several other genes (for example xenobiotic biodegradation and metabolism) showed in Fig. 2B, albeit with lesser contributions characterizing the group. Noteworthy, also fatty acid degradation gene abundances were observed to discriminate along the *x*-axes, which were found to be more prevalent in the HI0–HI25 samples, further underscoring the differential functional profiles between the two groups. No differences were highlighted in the biosynthesis of saturated and unsaturated fatty acid genes (Figure S2).

### Liver histology

**Fig. 3:**
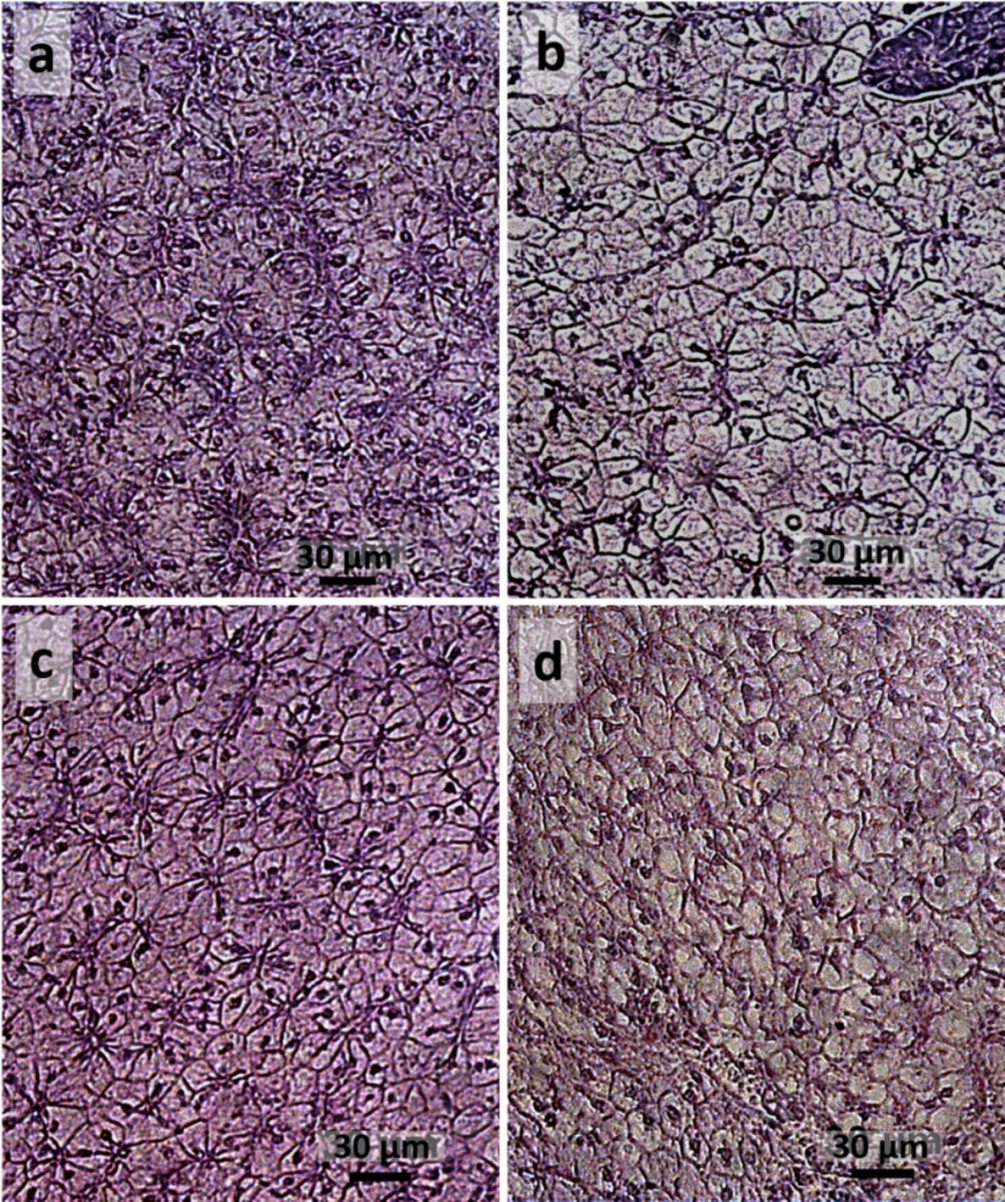
Representative histological pictures of liver from HI0 (**a**), HI25 (**b**), HI35 (**c**) and HI50 (**d**) groups. Hematoxylin and eosin. Scale bar = 30 µm

Representative histological pictures of liver from the different experimental groups are reported in Fig. 3. Results of quantitative and qualitative liver condition evaluations are summarized in Table 3. The number of hepatocytes per area analyzed was significantly higher in the HI0 group compared to HI50 one, while no significant differences were highlighted by the comparison with the other groups. In the liver from the HI25 group, a significantly higher incidence of granulocyte infiltration was highlighted compared to HI0 and HI35 groups. No other significant differences in the number of delocalized nuclei (peripheric nuclei) and in the other liver condition indexes were highlighted among the groups.

**Table 3:** Results from hepatocytes number and peripheral nuclei per area quantification and liver condition evaluation in terms of circulatory disturbance, regressive and progressive changes and granulocyte infiltration in liver from the different experimental groups. Data are reported as mean of the observation. Different letters indicate statistically significant differences among the groups (a,b; *p* < 0.05)

	HI0	HI25	HI35	HI50	*p-value*
Hepatocytes (n/0.3 mm^2^)	324.9 ± 34.6^a^	286.9 ± 33.7^ab^	271.1 ± 32.0^ab^	263.7 ± 32.4^b^	*0.029*
Peripheral nuclei (n/0.3 mm^2^)	277.6 ± 37.4	253.9 ± 31.2	241.5 ± 26.7	227.8 ± 34.9	*0.101*
Circulatory disturbance (score)	1.45 ± 0.37	1.75 ± 0.94	0.67 ± 0.88	2.04 ± 1.08	*0.068*
Regressive changes (score)	1.90 ± 0.42	2.17 ± 0.52	1.50 ± 0.55	2.29 ± 0.57	*0.068*
Progressive changes (score)	1.80 ± 0.67	2.17 ± 0.52	1.58 ± 0.58	1.64 ± 0.63	*0.347*
Granulocyte infiltration (score)	0.650 ± 0.602^b^	2.000 ± 0.791^a^	0.583 ± 0.563^b^	1.107 ± 0.453^ab^	*0.003*

## Discussions

The effects of the diets used in our research on gilthead seabream growth performances were previously assessed in a study from Di Rosa and colleagues (Di Rosa et al 2023), showing a marginal effect only.

In terms of microbiome composition, our analyses showed a high homogeneity within the samples extracted from tissues and those extracted from contents and scraping, suggesting that despite the efforts to distinguish them during the sampling, it remains challenging to analyze the transient microbiome separately from the resident microbiome (Tarnecki et al. 2017). The transient microbial community is primarily affected by environmental conditions and the host’s feeding habits, since it is composed of free-living microorganisms that enter the host’s body along with the water or feed and are soon expelled (Moschos et al. 2022).

At the end of the experiment, all the test diets induced a higher alpha diversity compared to the beginning (T0), with higher diversity values in HI35 and HI50 compared to HI0 and HI25 groups. A higher microbial richness should always be considered a positive effect, since it may potentially provide further metabolic capabilities to the host, thus improving its general health conditions (Borrelli et al. 2017). Busti and colleagues (2024) reported a decrease in alpha diversity in gut microbiome from juvenile gilthead seabream fed with 5 to 15% HI dietary inclusion, compared to a control FM-based diet, while no differences were reported in response to 30% and 35% FM substitution with HI (Panteli et al. 2021; Rimoldi et al. 2024). Our results indicate a possible role of HI percentage in increasing alpha diversity in the gut microbiome of gilthead seabream; although, other studies performed in different conditions (replacement percentage, duration and size) have shown discordant results, highlighting how the microbial composition is certainly influenced by the combination of several factors (Rimoldi et al. 2024; Quero et al. 2023; Busti et al. 2024).

According to recent research on seabream (Busti et al. 2024; Panteli et al. 2021), Proteobacteria, Actinobacteria and Firmicutes are the predominant taxa populating gut microbiome, with Firmicutes exhibiting the largest proportions, regardless of the diet used (Panteli et al. 2021). Both Panteli et al. (2021) and Rimoldi et al. (2024) reported lower relative abundances of Firmicutes in seabream fed on HI-based diets, compared to those fed a control diet in which FM was used as the only protein source. A decrease in the relative abundance of Firmicutes was also evidenced in response to FM replacement by different sources of protein in the diet in the same fish species (Estruch et al. 2015) and hypothesized to be related to the different fatty acid profile of the two ingredients. Similarly, in our study, we report that Firmicutes, which were not among the most prevalent phyla, generally decreased as FM replacement by HI increased in the diet.

Among the most abundant taxa identified in our results, Alphaproteobacteria, Actinobacteriota and Cyanobacteria were prevailing. Alphaproteobacteria were predominantly composed of ASVs from the family Rhizobiaceae, which were notably enriched in the HI0 and HI25 treatments. Rhizobiaceae are primarily known for containing anaerobic nitrogen–fixing bacteria (Lindström and Mousavi 2020) and for being present in recirculation system waters, but members of the Rhizobiaceae family were abundant in the intestinal microbiota of fish fed with the antibiotic florfenicol (FF) (Gupta et al. 2019). Members of the Rhizobiaceae family have been generally considered to be beneficial in other fish species such as Nile tilapia (Xia et al. 2018) and Atlantic salmon (Hartviksen et al. 2014) and hypothesized to have a positive role in nitrogen metabolism as well as in the removal of potential toxic molecules (Green et al. 2024). In more detail, the genus Aurantimonas, which was found at high abundances among all samples, has been primarily reported in soil and water; nevertheless, its presence has been previously observed in fish gut and linked to transient ingestion (Rimoldi et al. 2020). Recently, this genus was found as a core microbiome member in wild-caught seabream (Meriggi et al. 2024), suggesting a potential key role in this species’ microbiome. Also, in marine environment, phylogenetic groups from Alphaproteobacteria contributes to the uptake of low molecular compounds such as amino acids and protein playing, and a possible role in assimilating nutrients from feed cannot be ruled out (Cottrell and Kirchman 2000; Yokokawa and Nagata 2010).

On the other hand, microbiome from HI35 to HI50 groups was markedly characterized by high abundances of the Chloroflexi phylum, primarily represented by the classes Anaerolineae and Dehalococcoidia. Chloroflexi is a widespread and metabolically diverse phylum of bacteria, common in biofloc, water recirculation systems and other wastewater management systems (Petriglieri et al. 2023), where it has been reported to be involved in organic matter degradation processes (Guan et al. 2015; Almeida et al. 2021; Chen et al. 2024). To the best of our knowledge, direct evidence linking Dehalococcoidia and Anaerolineae to increased protein synthesis in fish appears to be limited or lacking in the current scientific literature. However, the presence of these members of the Chloroflexi phylum in the gut microbiome might be imputable to the transient microbiota and linked to a medium–high inclusion of HI in the diet. Considering that this result relies on a prediction of functional profiles based on 16S rRNA gene sequences, further investigations are required to evaluate the possible contribution of this taxon to fish health.

Remarkably, this phylum has been recently found as part of microbiota in gut of different cultured fish species, including gilthead seabream (Liu et al. 2021; Nikouli et al. 2021; Ruiz et al. 2023a, b) and other captive bred fish. As an example, Chloroflexi were found in domesticated zebrafish raised in indoor laboratory systems, but not in wild type (Pham et al. 2008), and also in wild caught mullets (*Chelon ramada*) (Le and Wang 2020; Floris et al. 2024). However, the impact of this phylum on fish physiology still remains largely unknown. In gilthead seabream, increased Chloroflexi abundance was previously correlated with increased gut mucous production, which, in turn, may have a further role in favoring gut colonization by these specific taxa (Naya-Català et al. 2021). Interestingly, in a previous study, an increase in mucous cell number and in mucosal folds width was observed in the intestine of gilthead seabream fed the same HI35 diet used in the present study, indicating an improved lubrication together with better absorptive mucosa condition in this group (Di Rosa et al. 2023). Thus, Chloroflexi may have had a pivotal role in improving gut condition in seabreams fed on medium–high HI percentage in the diets. Moreover, HI meal is known to act by selecting bacterial communities able to produce short-chain fatty acids (mainly butyrate) induced by chitin fermentation (Biasato et al. 2022; Rimoldi et al. 2019, 2021). Thus, a possible increase in Chloroflexi could be induced also by this factor, given that Choloflexi are also known as butyrate-oxidizing bacteria (Yi et al. 2020). Also, Chloroflexi, as well as Actinobacteria, were related to improved gut bacterial metabolic potentials involved in energy metabolism, carbohydrate metabolism, amino acid metabolism, environmental information processing and cellular processes in crucian carp (*Carassis auratus*) (Li et al. 2023a). The abundance of the phylums Chloroflexi and Actinobacteria was also positively correlated with improved growth in hybrid fish derived from herbivorous *Megalobrama amblycephala* (♀) × carnivorous *Culter alburnus* (♂) (Li et al. 2023b). In our study, Chloroflexi were remarkably represented in all the experimental groups, with a significant increase in HI35. As previously reported, the fatty acid profile of the HI35 diet used in the present study was dominated by lauric acid (C12:0) and palmitic acid (C16:0) (Oteri et al. 2021). Lauric acid (C12:0), particularly, is a short-medium chain FA, highly abundant in *H. illucens* meal and its role in exhibiting anti-inflammatory properties at intestinal level, and antimicrobial activity against Gram-positive bacteria has been widely demonstrated (Skrivanova et al. 2006; Spranghers et al. 2018; Vargas-Abúndez et al. 2019; Randazzo et al. 2023). Chloroflexi are mainly Gram-negative bacteria (Sutcliffe 2010), and the role of certain SCFAs could play a role in selecting microbiota communities, differently to what happens in marine wild fish populations, which feed on a varied diet, rich in long chain polyunsaturated fatty acids (LCPUFAs). Even though the role of Chloroflexi in fish gut is still unclear (Bovio et al. 2019), even a potential role in boosting fish detoxicant defense should be worthy of further investigations, since Chloroflexi have been shown to increase in gut microbiome of fish treated with different xenobiotics, such as microplastics (Zhang et al. 2024) and aromatic compounds (styrene and fluorobenzoate) (García-Márquez et al. 2022).

The result obtained using the PICRUSt-predicted metagenomes analysis can unveil functional redundancy across microbial communities, where different taxa perform similar ecological roles through convergent metabolic pathways (Louca et al. 2018). In this study, Cyanobacteria were observed across all the treatments, but in particular, showed a discriminant role for the HI0 and HI25 groups. This is in contrast with findings from Panteli and colleagues (2021), which found an increase in Cyanobacteria related with higher HI meal inclusion levels; unfortunately, is not possible to compare with other studies since Cyanobacteria sequences are often removed from the analysis (Rimoldi et al 2020). The presence and activity of Cyanobacteria were further supported by PICRUSt results, which identified genes associated with photosynthesis activity, even if, this function did not showed correlation with any specific treatment, suggesting that photosynthetic activity was not affected by the substitution of FM with HI. Predictive functional profiling of the microbial communities revealed slight differences in several metabolic pathways across the samples. Among all the predicted genes, a particular focus was paid towards the pathways associated with fatty acid metabolism and revealed a decrease in fatty acid conversion pathways corresponding to increasing HI levels. This result, coupled with the lack of differences in fatty acid biosynthesis, suggests a possible effect due to the different fatty acid profile in the diets (Oteri et al. 2021), resulting in selection of bacteria with a higher expression of FAs’ degradation pathway in HI0 and HI25 (with a higher percentage of FM in the diet) compared to HI35 and HI50 groups. Moreover, the HI35 and HI50 groups displayed increased activity in enzymatic pathways related to protein synthesis and translation, including ribosome and tRNA biosynthesis, leading to assume that protein synthesis was higher in gut microbiota from these groups. Chloroflexi, as the most representative phylum found in microbiota from HI35 to HI50 groups, are involved in several metabolic pathways in fish gut (Li et al. 2023b) and may be responsible of the results obtained by the PICRUSt analyses for these groups. However, further speculations cannot be done, since it is difficult to unambiguously identify bacterial strains responsible for a higher protein synthesis within the ones identified.

Overall, the adaptation of the microbial community to dietary changes point out to the importance of examining functional profiles, as they provide important insights into the metabolic adjustments and resilience of microbiomes to dietary interventions. It is important to note that the functional pathways were predicted using 16S rRNA gene sequencing data, and further functional validation is necessary.

Histological analyses highlighted a significantly higher granulocyte infiltration in liver from the HI25 group, compared to the other experimental groups. Granulocytes are present in blood and in a wide range of fish tissue and contain a high number of functional proteins, including antimicrobial peptides and enzymes with a crucial role in innate immune defenses (Lauriano et al. 2012; Cao et al. 2023). Since no histo-pathological signs or significantly appreciable morphological alterations were highlighted in the HI25 group, the increase in granulocytes in the liver from this fish could be not ascribable to an ongoing inflammatory process, but rather to enhanced innate defences in fish-fed HI meal low inclusion. This result can be described as a hormesis effect. A parallel adaptive physiological response was reported by Di Rosa and colleagues (Di Rosa et al. 2023), which observed a higher hepato-somatic index (HSI) and a lower viscero-somatic index (VSI) in the same fish-fed HI25 diet.

Hormesis is defined as an adaptive response to low-intensity/dose stimuli (Calabrese et al. 2007). An adaptive hormetic response was observed often in vertebrate models, including fish, subject to dietary and toxicant stimuli and was likely related to a triggering in immune system activity (Rix et al. 2022). On the other hand, significant results emerged by the analyses of hepatocytes number per area, which provide an estimation of hepatocytes number: the less they are for area, the bigger they are. The tendency to decrease hepatocytes number per area related to dietary HI meal inclusion which lead to statistically significant differences in HI50 group, compared to the HI0 one, indicates increased hepatocytes size, particularly in HI50 group, indicating a higher lipid deposition, tending to a liver steatotic condition. The liver plays a pivotal role in lipid metabolism and deposition, and its histological architecture is strictly dependent on lipid composition and profile of the diet. As previously mentioned, HI35 and HI50 diets were particularly rich in SCFAs compared to the other diets, as a consequence of a high HI meal inclusion (Oteri et al. 2021). A similar high lipid deposition in the liver was already reported in gilthead bream fed on a vegetable-based diet in which HI meal was used as protein source (40% of the dietary crude protein) and has been related to the HI lipid profile (Randazzo et al 2021). Compared to freshwater fish species, saltwater ones retain a lower ability in converting short-chain precursors in highly unsaturated FAs through the enzymatic elongation and desaturation pathways (Tocher 2010), which in turn may lead to a higher lipid deposition in liver parenchyma.

## Conclusions

Fish health is dependent on exogenous and endogenous factors. In the present study, the changes in the microbiome community related to high *Hermetia illucens* meal levels in the diet indicate a critical threshold, beyond whose bacterial community is significantly affected by the diet composition. Also, for the first time, the relevant presence of Chloroflexi in gilthead seabream opens cues on the role of this phylum in dietary adaptive response. Despite similar findings have been already observed in this species when fed *Hermetia illucens*-based diets, further investigations are required to evaluate the possible contribution of this taxon to fish health. Moreover, a pivotal role of diet fatty acid composition on liver lipid deposition was confirmed, suggesting that the tolerance of gilthead seabream to high percentage of *Hermetia illucens* in the diet is limited.

## Supplementary Information

Below is the link to the electronic supplementary material.

Supplementary file1 (DOCX 302 KB)

## Data Availability

No datasets were generated or analysed during the current study.

## References

[CR1] Abdel-Latif HMR, Abdel-Tawwab M, Khalil RH, Metwally AA, Shakweer MS, Ghetas HA, Khallaf MA (2021) Black soldier fly (*Hermetia**illucens*) larvae meal in diets of European seabass: effects on antioxidative capacity, non-specific immunity, transcriptomic responses, and resistance to the challenge with *Vibrio**alginolyticus*. Fish Shellfish Immunol 111:111–118. 10.1016/j.fsi.2021.01.013 33508473 10.1016/j.fsi.2021.01.013

[CR2] Abdel-Tawwab M, Khalil RH, Metwally AA, Shakweer MS, Khallaf MA, Abdel-Latif HMR (2020) Effects of black soldier fly (Hermetia illucens L.) larvae meal on growth performance, organs-somatic indices, body composition, and hemato-biochemical variables of European sea bass, Dicentrarchus labrax. Aquaculture 522:735136. 10.1016/j.aquaculture.2020.735136

[CR3] Abu Bakar N-H, Abdul Razak S, Mohd Taufek N, Alias Z (2021) Evaluation of black soldier fly (*Hermetia**illucens*) prepupae oil as meal supplementation in diets for red hybrid tilapia (*Oreochromis* sp.). Int J Trop Insect Sci 41:2093–2102. 10.1007/s42690-020-00398-z

[CR4] Adeoye AA, Akegbejo-Samsons Y, Fawole FJ, Davies SJ (2020) Preliminary assessment of black soldier fly (*Hermetia**illucens*) larval meal in the diet of African catfish (*Clarias**gariepinus*): impact on growth, body index, and hematological parameters. J World Aquacult Soc 51:1024–1033. 10.1111/jwas.12691

[CR5] Agbohessou PS, Mandiki SNM, Gougbédji A, Megido RC, Hossain MdS, De Jaeger P, Larondelle Y, Francis F, Lalèyè PA, Kestemont P (2021) Total replacement of fish meal by enriched-fatty acid *Hermetia**illucens* meal did not substantially affect growth parameters or innate immune status and improved whole body biochemical quality of Nile tilapia juveniles. Aquac Nutr 27:880–896. 10.1111/anu.13232

[CR6] Albrektsen S, Kortet R, Vilhelm Skov P, Ytterborg E, Gitlesen S, Kleinegris D, Mydland L-T, Hansen JØ, Lock E-J, Mørkøre T, James P, Wang X, Whitaker RD, Vang B, Hatlen B, Daneshvar E, Bhatnagar A, Jensen LB, Øverland M (2022) Future feed resources in sustainable salmonid production: a review. Rev Aquac 14(4):1790–1812. 10.1111/raq.12673

[CR7] Almeida DB, Magalhães C, Sousa Z, Borges MT, Silva E, Blanquet I, Mucha AP (2021) Microbial community dynamics in a hatchery recirculating aquaculture system (RAS) of sole (Solea senegalensis). Aquaculture 539:736592. 10.1016/j.aquaculture.2021.736592

[CR8] Antonopoulou E, Nikouli E, Piccolo G, Gasco L, Gai F, Chatzifotis S, Mente E, Kormas KA (2019) Reshaping gut bacterial communities after dietary *Tenebrio**molitor* larvae meal supplementation in three fish species. Aquaculture 503:628–635. 10.1016/j.aquaculture.2018.12.013

[CR9] Aragão C, Cabano M, Colen R, Fuentes J, Dias J (2022) Alternative formulations for gilthead seabream diets: towards a more sustainable production. Aquac Nutr 26:444–455. 10.1111/anu.13007

[CR10] Bakke AM, Glover C, Krogdahl Å (2010) Feeding, digestion and absorption of nutrients. In: Grosell M, Farrell AP, Brauner CJ (eds) Fish physiology. Elsevier: Amsterdam, The Netherlands 30, 57–110. 10.1016/S1546-5098(10)03002-5

[CR11] Barragan-Fonseca KB, Dicke M, van Loon JJA (2017) Nutritional value of the black soldier fly (*Hermetia**illucens* L.) and its suitability as animal feed - a review. J Insects Food Feed 3:105–120. 10.3920/jif2016.0055

[CR12] Barroso FG, de Haro C, Sánchez-Muros MJ, Venegas E, Martínez-Sánchez A, Pérez-Bañón C (2014) The potential of various insect species for use as food for fish. Aquaculture 422:193–201. 10.1016/j.aquaculture.2013.12.024

[CR13] Basto A, Matos E, Valente LM (2020) Nutritional value of different insect larvae meals as protein sources for European sea bass (*Dicentrarchus labrax*) juveniles. Aquaculture 521:735085. 10.1016/j.aquaculture.2020.735085

[CR14] Benhabiles MS, Salah R, Lounici H, Drouiche N, Goosen MFA, Mameri N (2012) Antibacterial activity of chitin, chitosan and its oligomers prepared from shrimp shell waste. Food Hydrocoll 29:48–56. 10.1016/j.foodhyd.2012.02.013

[CR15] Bernet D, Schmidt H, Meier W, Burkhardt-Holm P, Wahli T (1999) Histopathology in fish: proposal for a protocol to assess aquatic pollution. J Fish Dis 22:25–34. 10.1046/j.1365-2761.1999.00134.x

[CR16] Biasato I, Chemello G, Oddon SB, Ferrocino I, Corvaglia MR, Caimi C, Resconi A, Paul A, van Spankeren M, Capucchio MT, Colombino E, Cocolin L, Gai F, Schiavone A, Gasco L (2022) *Hermetia illucens* meal inclusion in low-fishmeal diets for rainbow trout (*Oncorhynchus mykiss*): effects on the growth performance, nutrient digestibility coefficients, selected gut health traits, and health status indices. Anim Feed Sci Technol 290:115341. 10.1016/j.anifeedsci.2022.115341

[CR17] Borrelli L, Coretti L, Dipineto L, Bovera F, Menna F, Chiariotti L, Nizza A, Lembo F, Fioretti A (2017) Insect-based diet, a promising nutritional source, modulates gut microbiota composition and SCFAs production in laying hens. Sci Rep 7(1):16269. 10.1038/s41598-017-16560-6 29176587 10.1038/s41598-017-16560-6 PMC5701250

[CR18] Borrelli L, Varriale L, Dipineto L, Pace A, Menna LF, Fioretti A (2021) Insect derived lauric acid as promising alternative strategy to antibiotics in the antimicrobial resistance scenario. Front Microbiol 12:620798. 10.3389/FMICB.2021.620798 33717009 10.3389/fmicb.2021.620798 PMC7952302

[CR19] Bovio P, Cabezas A, Etchebehere C (2019) Preliminary analysis of *Chloroflexi* populations in full-scale UASB methano-genic reactors. J Appl Microbiol 126(2):667–683. 10.1111/jam.14115 30269410 10.1111/jam.14115

[CR20] Bruni L, Belghit I, Lock E-J, Secci G, Taiti C, Parisi G (2020a) Total replacement of dietary fish meal with black soldier fly (*Hermetia**illucens*) larvae does not impair physical, chemical or volatile composition of farmed Atlantic salmon (*Salmo**salar* L.). J Sci Food Agric 100:1038–1047. 10.1002/jsfa.10108 31650558 10.1002/jsfa.10108

[CR21] Bruni L, Randazzo B, Cardinaletti G, Zarantoniello M, Mina F, Secci G, Tulli F, Olivotto I, Parisi G (2020b) Dietary inclusion of full-fat *Hermetia**illucens* prepupae meal in practical diets for rainbow trout (*Oncorhynchus**mykiss*): lipid metabolism and fillet quality investigations. Aquaculture 529:735678. 10.1016/j.aquaculture.2020.735678

[CR22] Bruslé J, Gonzàlez I, Anadon G (2017) The structure and function of fish liver. In: Datta Munshi JS, Dutta HM (eds) Fish morphology. Routledge: London, UK, pp 77–93. 10.1201/9780203755990-6

[CR23] Busti S, Bonaldo A, Candela A, Scicchitano D, Trapella G, Brambilla F, Guidou C, Trespeuch C, Sirri F, Dondi F, Gatta PP, Parma L (2024) *Hermetia**illucens* larvae meal as an alternative protein source in practical diets for gilthead sea bream (*Sparus**aurata*): a study on growth, plasma biochemistry and gut microbiota. Aquaculture 578:740093. 10.1016/j.aquaculture.2023.740093

[CR24] Caimi C, Biasato I, Chemello G, Oddon SB, Lussiana C, Malfatto VM, Capucchio MT, Colombino E, Schiavone A, Gai F, Trocino A, Brugiapaglia A, Renna M, Gasco L (2021) Dietary inclusion of a partially defatted black soldier fly (*Hermetia**illucens*) larva meal in low fishmeal-based diets for rainbow trout (*Oncorhynchus**mykiss*). J Anim Sci Biotechnol 12:50. 10.1186/s40104-021-00575-1 33858519 10.1186/s40104-021-00575-1 PMC8050899

[CR25] Calabrese EJ, Bachmann KA, Bailer AJ, Bolger PM, Borak J, Cai L, Cedergreen N, Cherian MG, Chiueh CC, Clarkson TW, Cook RR, Diamond DM, Doolittle DJ, Dorato MA, Duke SO, Feinendegen L, Gardner DE, Hart RW, Hastings KL, Hayes AW, Hoffmann GR, Ives JA, Jaworowski Z, Johnson TE, Jonas WB, Kaminski NE, Keller JG, Klaunig JE, Knudsen TB, Kozumbo WJ, Lettieri T, Liu SZ, Maisseu A, Maynard KI, Masoro EJ, McClellan RO, Mehendale HM, Mothersill C, Newlin DB, Nigg HN, Oehme FW, Phalen RF, Philbert MA, Rattan SI, Riviere JE, Rodricks J, Sapolsky RM, Scott BR, Seymour C, Sinclair DA, Smith-Sonneborn J, Snow ET, Spear L, Stevenson DE, Thomas Y, Tubiana M, Williams GM, Mattson MP (2007) Biological stress response terminology: integrating the concepts of adaptive response and preconditioning stress within a hormetic dose-response framework. Toxicol Appl Pharmacol 222(1):122–128. 10.1016/j.taap.2007.02.015 17459441 10.1016/j.taap.2007.02.015

[CR26] Callahan BJ, McMurdie PJ, Rosen MJ, Han AW, Johnson AJ, Holmes SP (2016) DADA2: high-resolution sample inference from Illumina amplicon data. Nat Methods 13:581–583. 10.1038/nmeth.3869 27214047 10.1038/nmeth.3869 PMC4927377

[CR27] Cao J, Kong W, Cheng G, Xu Z (2023) Role of mTORC1 signaling in regulating the immune function of granulocytes in teleost fish. Int J Mol Sci 24(18):13745. 10.3390/ijms241813745 37762047 10.3390/ijms241813745 PMC10530975

[CR28] Carvalho M, Torrecillas S, Montero D, Sanmartín A, Fontanillas R, Farías A, Moutou K, Velásquez JH, Izquierdo M (2023) Insect and single-cell protein meals as replacers of fish meal in low fish meal and fish oil diets for gilthead sea bream (*Sparus**aurata*) juveniles. Aquaculture 566:739215. 10.1016/j.aquaculture.2022.739215

[CR29] Chen C, Yang Y, Graham NJ, Li Z, Yang X, Wang Z, Farhat N, Vrouwenvelder JS, Hou LA (2024) A comprehensive evaluation of the temporal and spatial fouling characteristics of RO membranes in a full-scale seawater desalination plant. Water Res 249:120914. 10.1016/j.watres.2023.120914 38007899 10.1016/j.watres.2023.120914

[CR30] Collins SA, Øverland M, Skrede A, Drew MD (2013) Effect of plant protein sources on growth rate in salmonids: meta-analysis of dietary inclusion of soybean, pea and canola/rapeseed meals and protein concentrates. Aquaculture 400:85–100. 10.1016/j.aquaculture.2005.10.032

[CR31] Cottrell MT, Kirchman DL (2000) Natural assemblages of marine proteobacteria and members of the Cytophaga-Flavobacter cluster consuming low- and high-molecular-weight dissolved organic matter. Appl Environ Microbiol 66:1692–1697. 10.1128/aem.66.4.1692-1697.2000 10742262 10.1128/aem.66.4.1692-1697.2000 PMC92043

[CR32] Di Rosa AR, Caccamo L, Pansera L, Oteri M, Chiofalo B, Maricchiolo G (2023) Influence of *Hermetia**illucens* larvae meal dietary inclusion on growth performance, gut histological traits and stress parameters in *Sparus**aurata*. Animals 13:339. 10.3390/ani13030339 36766228 10.3390/ani13030339 PMC9913394

[CR33] Donadelli V, Di Marco P, Mandich A, Finoia MG, Cardinaletti G, Petochi T, Longobardi A, Tibaldi E, Marino G (2024) Effects of dietary plant protein replacement with insect and poultry by-product meals on the liver health and serum metabolites of sea bream (*Sparus**aurata*) and sea bass (*Dicentrarchus**labrax*). Animals (Basel) 14(2):241. 10.3390/ani14020241 38254412 10.3390/ani14020241 PMC10812684

[CR34] Estruch G, Collado MC, Peñaranda DS, Tomás Vidal A, Jover Cerdá M, Perez Martìnez S, Martinez-Llorens S (2015) Impact of fishmeal replacement in diets for gilthead sea bream (*Sparus**aurata*) on the gastrointestinal microbiota determined by pyrosequencing the 16S rRNA Gene. PLoS ONE 10(8):e0136389. 10.1371/journal.pone.0136389 26317431 10.1371/journal.pone.0136389 PMC4552794

[CR35] European Directive (2010) European Directive 2010/63/EU of the European Parliament and of the Council of 22 September 2010 on the protection of animals used for scientific purposes. Off J Eur 276:33–79

[CR36] European Directive (2014) Legislative Decree No. 26 implementing European Directive 2010/63/EU of the European Parliament and of the Council of 22 September 2010 on the protection of animals used for scientific purposes. Off J Ital Leg 61:1–36

[CR37] Fabrikov D, Vargas-García MdC, Barroso FG, Sànchez-Muros MJ, Cacua Ortíz SM, Morales AE, Cardenete G, Tomás-Almenar C, Melenchón F (2021) Effect on intermediary metabolism and digestive parameters of the high substitution of fishmeal with insect meal in *Sparus**aurata* feed. Insects 12(11):965. 10.3390/insects12110965 34821766 10.3390/insects12110965 PMC8618839

[CR38] FAO (2022) The state of world fisheries and aquaculture 2022. Towards Blue Transformation; FAO: Rome, Italy, 2022

[CR39] Fawole FJ, Adeoye AA, Tiamiyu LO, Ajala KI, Obadara SO, Ganiyu IO (2020) Substituting fishmeal with *Hermetia**illucens* in the diets of African catfish (*Clarias**gariepinus*): effects on growth, nutrient utilization, haemato-physiological response, and oxidative stress biomarker. Aquaculture 518:734849. 10.1016/j.aquaculture.2019.734849

[CR40] FEAP (2023) Federation of European Aquaculture Producers Report

[CR41] Ferosekhan S, Xu H, Turkmen S, Gómez A, Afonso JM, Fontanillas R, Rosenlund G, Kaushik S, Izquierdo M (2020) Reproductive performance of gilthead seabream (*Sparus**aurata*) broodstock showing different expression of fatty acyl desaturase 2 and fed two dietary fatty acid profiles. Sci Rep 10:1–14. 10.1038/s41598-020-72166-5 32968090 10.1038/s41598-020-72166-5 PMC7512018

[CR42] Fisher HJ, Collins SA, Hanson C, Mason B, Colombo SM, Anderson DM (2020) Black soldier fly larvae meal as a protein source in low fish meal diets for Atlantic salmon (*Salmo**salar*). Aquaculture 521:734978. 10.1016/j.aquaculture.2020.734978

[CR43] Floris R, Sanna G, Teodora SC, Battaggia G, Pascale FD, Vezzi A, Fois N (2024) Microbial communities associated with the intestinal tract of grey mullets from a Mediterranean aquatic environment. Preprints 2024051532. 10.20944/preprints202405.1532.v1

[CR44] Gai F, Cusimano G, Maricchiolo G, Caccamo L, Caimi C, Macchi E, Meola M, Perdichizzi A, Tartarisco G, Gasco L (2023) Defatted black soldier fly meal in diet for grow-out gilthead seabream (Sparus aurata L. 1758): effects on growth performance, gill cortisol level, digestive enzyme activities, and intestinal histological structure. Aquac Res 2(1–18):1–18. 10.1155/2023/3465335

[CR45] Galkanda-Arachchige HSC, Wilson AE, Davis DA (2020) Success of fishmeal replacement through poultry by-product meal in aquaculture feed formulations: a meta-analysis. Rev Aquac 12:1624–1636. 10.1111/raq.12401

[CR46] García-Márquez J, Cerezo IM, Figueroa LF, Abdala-Díaz RT, Arijo S (2022) First evaluation of associated gut micro-biota in wild thick-lipped grey mullets (*Chelon**labrosus*, Risso 1827). Fishes 7:209. 10.3390/fishes7040209

[CR47] Gasco L, Gai F, Maricchiolo G, Genovese L, Sergio R, Bottari T, Caruso G (2018) Fishmeal alternative protein sources for aquaculture feeds. Chapter in: Feeds for the aquaculture sector. Springer, Cham. 10.1007/978-3-319-77941-61

[CR48] Gatlin DM III, Barrows FT, Brown P, Dabrowski K, Gaylord TG, Hardy RW, Herman E, Hu G, Krogdahl Å, Nelson R, Overturf K (2007) Expanding the utilization of sustainable plant products in aquafeeds: a review. Aquac Res 38(6):551–79. 10.1111/j.1365-2109.2007.01704.x

[CR49] Glencross BD (2009) Exploring the nutritional demand for essential fatty acids by aquaculture species. Rev Aquacult 1:71–124. 10.1111/j.1753-5131.2009.01006.x

[CR50] Green GB, Williams MB, Brandom JL, Chehade SB, Fay CX, Morrow CD et al (2024) A bacterial-sourced protein diet induces beneficial shifts in the gut microbiome of the zebrafish, Danio Rerio. Curr Develop Nutrition 8:102077. 10.1016/j.cdnut.2024.102077 10.1016/j.cdnut.2024.102077 PMC10865222 38357379

[CR51] Guan W, Yin M, He T, Xie S (2015) Influence of substrate type on microbial community structure in vertical-flow constructed wetland treating polluted river water. Environ Sci Pollut Res 22:16202–16209. 10.1007/s11356-015-5160-9 10.1007/s11356-015-5160-9 26263887

[CR52] Guerreiro I, Serra CR, Coutinho F, Couto A, Castro C, Rangel F, Peres H, Pousao-Ferreira P, Matos E, Gasco L, Gai F, Oliva-Teles A, Enes P (2021) Digestive enzyme activity and nutrient digestibility in meagre (*Argyrosomus**regius*) fed increasing levels of black soldier fly meal (*Hermetia**illucens*). Aquac Nutr 27:142–152. 10.1111/anu.13172

[CR53] Gupta S, Fernandes J, Kiron V (2019) Antibiotic-induced perturbations are manifested in the dominant intestinal bacterial phyla of Atlantic salmon. Microorganisms 7(8):233. 10.3390/microorganisms7080233 31382431 10.3390/microorganisms7080233 PMC6723382

[CR54] Hartviksen M, Vecino J, Ringø E, Bakke AM, Wadsworth S, Krogdahl Å, Ruohonen K, Kettunen A (2014) Alternative dietary protein sources for Atlantic salmon (*Salmo**salar* L.) effect on intestinal microbiota, intestinal and liver histology and growth. Aquac Nutr 20:381–398. 10.1111/anu.12087

[CR55] Hasan I, Rimoldi S, Saroglia G, Terova G (2023) Sustainable fish feeds with insects and probiotics positively affect freshwater and marine fish gut microbiota. Animals 13:1633. 10.3390/ani13101633 37238063 10.3390/ani13101633 PMC10215438

[CR56] Hender A, Siddik MA, Howieson J, Fotedar R (2021) Black soldier fly, *Hermetia illucens* as an alternative to fishmeal protein and fish oil: impact on growth, immune response, mucosal barrier status, and flesh quality of juvenile barramundi, *Lates**calcarifer* (Bloch, 1790). Biology 10(6):505. 10.3390/biology10060505 34200162 10.3390/biology10060505 PMC8230191

[CR57] Henry MA, Gasco L, Piccolo G, Fontoulaki E (2015) Review on the use of insects in the diet of farmed fish: past and future. JAFST 203:1–22. 10.1016/j.anifeedsci.2015.03.001

[CR58] Hilborn R, Banobi J, Hall SJ, Pucylowski T, Walsworth TE (2018) The environmental cost of animal source foods. Front Ecol Environ 16:329–335. 10.1002/fee.1822

[CR59] Hoc B, Genva M, Fauconnier ML, Lognay G, Francis F, Caparros Megido R (2020) About lipid metabolism in Hermetia illucens (L. 1758): on the origin of fatty acids in prepupae. Sci Rep 10(1):11916. 10.1038/S41598-020-68784-8 32680992 10.1038/s41598-020-68784-8 PMC7368053

[CR60] Hoc B, Tomson T, Malumba P, Blecker C, Jijakli MH, Purcaro G, Francis F, Caparros Megido R (2021) Production of rainbow trout (*Oncorhynchus**mykiss*) using black soldier fly (*Hermetia**illucens*) prepupae-based formulations with differentiated fatty acid profiles. Sci Total Environ 794:148647. 10.1016/j.scitotenv.2021.148647 34217091 10.1016/j.scitotenv.2021.148647

[CR61] Hoseinifar SH, Ashouri G, Marisaldi L, Candelma M, Basili D, Zimbelli A, Notarstefano V, Salvini L, Randazzo B, Zarantoniello M, Pessina A, Sojan JM, Vargas A, Carnevali O (2024) Reducing the use of antibiotics in European aquaculture with vaccines, functional feed additives and optimization of the gut microbiota. J Mar Sci Eng 12:204. 10.3390/jmse12020204

[CR62] Hossain MS, Small BC, Hardy R (2023) Insect lipid in fish nutrition: recent knowledge and future application in aquaculture. Rev Aquac 15(4):1664–1685. 10.1111/raq.12810

[CR63] Howlader S, Sumi KR, Sarkar S, Billah SM, Ali ML, Howlader J, Shahjahan M (2023) Effects of dietary replacement of fish meal by soybean meal on growth, feed utilization, and health condition of stinging catfish, *Heteropneustes**fossilis*. Saudi J Biol Sci 30(3):103601. 10.1016/j.sjbs.2023.103601 36874199 10.1016/j.sjbs.2023.103601 PMC9982026

[CR64] Hua K, Cobcroft JM, Cole A, Condon K, Jerry DR, Mangott A, Praeger C, Vucko MJ, Zeng C, Zenger K, Strungnell JM (2019) The future of aquatic protein: implications for protein sources in aquaculture diets. One Hearth 1(3):316–329. 10.1016/j.oneear.2019.10.018

[CR65] Huyben D, Vidakovic A, Werner Hallgren S, Langeland M (2019) High-throughput sequencing of gut microbiota in rainbow trout (*Oncorhynchus**mykiss*) fed larval and pre-pupae stages of black soldier fly (*Hermetia**illucens*). Aquaculture 500:485–491. 10.1016/j.aquaculture.2018.10.034

[CR66] Izquierdo MS, Robaina L, Juárez-Carrillo E, Oliva V, Hernández-Cruz CM, Afonso JM (2008) Regulation of growth, fatty acid composition and delta 6 desaturase expression by dietary lipids in gilthead seabream larvae (*Sparus**aurata*). Fish Physiol Biochem 34:117–127. 10.1007/s10695-007-9152-7 18649029 10.1007/s10695-007-9152-7

[CR67] Jannathulla R, Rajaram V, Kalanjiam R, Ambasankar K, Muralidhar M, Daval J (2019) Fishmeal availability in the scenarios of climate change: Inevitability of fishmeal replacement in aquafeeds and approaches for the utilization of plant protein sources. Aquac Res 50:3493–3506. 10.1111/are.14324

[CR68] Karapanagiotidis IT, Neofytou MC, Asimaki A, Daskalopoulou E, Psofakis P, Mente E, Rumbos CI, Athanassiou CG (2023) Fishmeal replacement by full-fat and defatted *Hermetia**illucens* prepupae meal in the diet of gilthead seabream (*Sparus**aurata*). Sustainability 15:786. 10.3390/su15010786

[CR69] Klindworth A, Pruesse E, Schweer T, Peplies J, Quast C, Horn M, Glöckner FO (2013) Evaluation of general 16S ribosomal RNA gene PCR primers for classical and next-generation sequencing-based diversity studies. Nucleic Acids Res 41(1):e1–e1. 10.1093/nar/gks808 22933715 10.1093/nar/gks808 PMC3592464

[CR70] Komi DEA, Sharma L, Cruz CSD (2018) Chitin and its effects on inflammatory and immune responses. Clin Rev Allergy Immunol 54(2):213–223. 10.1007/s12016-017-8600-0 28251581 10.1007/s12016-017-8600-0 PMC5680136

[CR71] Langille MG, Zaneveld J, Caporaso JG, McDonald D, Knights D, Reyes JA, Clemente JC, Burkepile DE, Vega Thurber RL, Knight R, Beiko RG, Huttenhower C (2013) Predictive functional profiling of microbial communities using 16S rRNA marker gene sequences. Nat Biotechnol 31(9):814–821. 10.1038/nbt.2676 23975157 10.1038/nbt.2676 PMC3819121

[CR72] Lauriano ER, Calò M, Silvestri G, Zaccone D, Pergolizzi S, Cascio PL (2012) Mast cells in the intestine and gills of the sea bream, *Sparus**aurata*, exposed to a polychlorinated biphenyl, PCB 126. Acta Histochem 114(2):166–171. 10.1016/j.acthis.2011.04.004 21565388 10.1016/j.acthis.2011.04.004

[CR73] Le MH, Wang D (2020) Structure and membership of gut microbial communities in multiple fish cryptic species under potential migratory effects. Sci Rep 10:7547. 10.1038/s41598-020-64570-8 32372020 10.1038/s41598-020-64570-8 PMC7200715

[CR74] Li S, Mai K, Xu W, Yuan Y, Zhang Y, Ai Q (2014) Characterization, mRNA expression and regulation of δ6 fatty acyl desaturase (FADS2) by dietary n-3 long chain polyunsaturated fatty acid (LC-PUFA) levels in grouper larvae (*Epinephelus**coioides*). Aquaculture 434:212–219. 10.1016/j.aquaculture.2014.08.009

[CR75] Li Y, Kortner TM, Chikwati EM, Belghit I, Lock E-J, Krogdahl Å (2020) Total replacement of fish meal with black soldier fly (*Hermetia**illucens*) larvae meal does not compromise the gut health of Atlantic salmon (*Salmo**salar*). Aquaculture 520:734967. 10.1016/j.aquaculture.2020.734967

[CR76] Li S, Gu Q, Li Z, Zeng Q, Zhong H, Liu M, Chen J, Zhou Y, Liu S, Hu S (2023a) The effects of lotus-fish co-culture on the gut microbiome of Hefang crucian carp (Carassis auratus). Reprod Breed 3(3):143–151. 10.1016/j.repbre.2023.09.001

[CR77] Li W, Zhou Z, Li H, Wang S, Ren L, Hu J, Liu Q, Wu C, Tang C, Hu F, Zeng L, Zhao R, Tao M, Zhang C, Qin Q, Liu S (2023b) Successional changes of microbial communities and host-microbiota interactions contribute to dietary adaptation in allodiploid hybrid fish. Microb Ecol 85:1190–1201. 10.1007/s00248-022-01993-y 35366074 10.1007/s00248-022-01993-y

[CR78] Lindström K, Mousavi SA (2020) Effectiveness of nitrogen fixation in rhizobia. Microb Biotechnol 13(5):1314–1335. 10.1111/1751-7915.13517 31797528 10.1111/1751-7915.13517 PMC7415380

[CR79] Liu T, Han T, Wang J, Liu T, Bian P, Wang Y, Cai X (2021) Effects of replacing fish meal with soybean meal on growth performance, feed utilization and physiological status of juvenile redlip mullet *Liza**haematocheila*. Aquac Rep 20:100756. 10.1016/j.aqrep.2021.100756

[CR80] Lomex RG, Hahs-Vaughn DL (2012) Statistical concepts: a second course, 4th edn. Routledge, New York, p 21. 10.4324/9780203137802

[CR81] Louca S, Polz MF, Mazel F, Albright MB, Huber JA, O’Connor MI, Ackermann M, Hahn AS, Srivastava DS, Crowe SA, Doebeli M, Parfrey LW (2018) Function and functional redundancy in microbial systems. Nat Ecol Evol 2(6):936–943. 10.1038/s41559-018-0519-1 29662222 10.1038/s41559-018-0519-1

[CR82] Macusi ED, Cayacay MA, Borazon EQ, Sales AC, Habib A, Fadli N, Santos MD (2023) Protein fishmeal replacement in aquaculture: a systematic review and implications on growth and adoption viability. Sustainability 15:12500. 10.3390/su151612500

[CR83] Magalhães R, Sanchez-Lopez A, Leal RS, Martínez-Llorens S, Oliva-Teles A, Peres H (2017) Black soldier fly (*Hermetia**illucens*) pre-pupae meal as a fish meal replacement in diets for European seabass (*Dicentrarchus**labrax*). Aquaculture 476:79–85. 10.1016/j.aquaculture.2017.04.021

[CR84] Maiolo S, Parisi G, Biondi N, Lunelli F, Tibaldi E, Pastres R (2020) Fishmeal partial substitution within aquafeed formulations: life cycle assessment of four alternative protein sources. Int J Life Cycle Assess 25:1455–1471. 10.1007/s11367-020-01759-z

[CR85] Martin M (2011) Cutadapt removes adapter sequences from high-throughput sequencing reads. EMBnet J 17:10–12. 10.14806/ej.17.1.200

[CR86] Maulu S, Langi S, Hasimuna OJ, Missinhoun D, Munganga BP, Hampuwo BM, Gabriel NN, Elsabagh M, Van Doan H, Abdul Kari Z, Dawood MAO (2022) Recent advances in the utilization of insects as an ingredient in aquafeeds: a review. Anim Nutr 11:334–349. 10.1016/j.aninu.2022.07.013 36329686 10.1016/j.aninu.2022.07.013 PMC9618972

[CR87] Maxwell SE, Delaney HD, Kelley K (2018) Designing experiments and analyzing data: a model comparison perspective, third ed. Routledge, New York, USA, p. 223. 10.4324/9781315642956

[CR88] McMurdie PJ, Holmes S (2013) phyloseq: an R package for reproducible interactive analysis and graphics of microbiome census data. PLoS ONE 8(4):e61217. 10.1371/journal.pone.0061217 23630581 10.1371/journal.pone.0061217 PMC3632530

[CR155] Meriggi N, Russo A, Renzi S, Cerasuolo B, Nerini M, Ugolini A, Marvasi M, Cavalieri D (2024) Enhancing seafood traceability: tracking the origin of seabass and seabream from the tuscan coast area by the analysis of the gill bacterial communities. Anim Microbiome 6(1):13. 10.1186/s42523-024-00300-z 10.1186/s42523-024-00300-z PMC10938666 38486253

[CR89] Moschos S, Kormas KA, Karayanni H (2022) Prokaryotic diversity in marine and freshwater recirculating aquaculture systems. Rev Aquac 14(4):1861–1886. 10.1111/raq.12677

[CR90] Moutinho S, Pedrosa R, Magalhaes R, Oliva-Teles A, Parisi G, Peres H (2021) Black soldier fly (*Hermetia**illucens*) pre-pupae larvae meal in diets for European seabass (*Dicentrarchus**labrax*) juveniles: effects on liver oxidative status and fillet quality traits during shelf-life. Aquaculture 533(736):080. 10.1016/j.aquaculture.2020.736080

[CR91] National Research Council (NRC) (2012) Nutrient requirements of fish and shrimp. Aquac Int 20:601–602. 10.1007/s10499-011-9480-6

[CR92] Nawaz A, Javaid AB, Irshad S, Hoseinifar SH, Xionga H (2018) The functionality of prebiotics as immunostimulant: evidences from trials on terrestrial and aquatic animals. Fish Shell Immunol 76:272–278. 10.1016/j.fsi.2018.03.004 10.1016/j.fsi.2018.03.004 29510254

[CR93] Naya-Català F, do Vale PG, Piazzon MC, Fernandes AM, Calduch-Giner JA, Sitjà-Bobadilla A, Conceição LE, Pérez-Sánchez J (2021) Cross-talk between intestinal microbiota and host gene expression in gilthead sea bream (*Sparus**aurata*) juveniles: insights in fish feeds for increased circularity and resource utilization. Front Physiol 12:748265. 10.3389/fphys.2021.748265 34675821 10.3389/fphys.2021.748265 PMC8523787

[CR94] Naylor R, Fang S, Fanzo J (2023) A global view of aquaculture policy. Food Policy 116:102422. 10.1016/j.foodpol.2023.102422. (**Elsevier**)

[CR95] Nikouli E, Kormas KA, Jin Y, Olsen Y, Bakke I, Vadstein O (2021) Dietary lipid effects on gut microbiota of first feeding Atlantic salmon (Salmo salar L.). Front Mar Sci 8:665576. 10.3389/fmars.2021.665576

[CR96] Nugroho R, Nur M (2018) Insect-based protein: future promising protein source for fish cultured. IOP Conf Ser Earth Environ Sci 144:012002. 10.1088/1755-1315/144/1/012002

[CR97] Oksanen J, Blanchet FG, Kindt R, Legendre P, Minchin PR, O’hara RB, Simpson GL, Solymos P, Stevens MH, Wagner H (2013) Community ecology package. R package version 2(0)

[CR98] Olsen RL, Hasan MR (2012) A limited supply of fishmeal: impact on future increases in global aquaculture production. Trends Food Sci Technol 27:120–128. 10.1016/j.tifs.2012.06.003

[CR99] Oteri M, Di Rosa B, Lo Presti V, Giarratana F, Toscano G, Chiofalo B (2021) Black soldier fly larvae meal as alternative to fish meal for aquaculture feed. Sustainability 13(10):5447. 10.3390/su13105447

[CR100] Oteri M, Chiofalo B, Maricchiolo G, Toscano G, Nalbone L, Lo Presti V, Di Rosa AR (2022) Black soldier fly larvae meal in the diet of gilthead sea bream: effect on chemical and microbiological quality of filets. Front Nutr 9:896552. 10.3389/fnut.2022.896552 35685870 10.3389/fnut.2022.896552 PMC9172839

[CR101] Pahlow M, van Oel P, Mekonnen M, Hoekstra A (2015) Increasing pressure on freshwater resources due to terrestrial feed ingredients for aquaculture production. Sci Total Environ 536:847–857. 10.1016/j.scitotenv.2015.07.124 26258557 10.1016/j.scitotenv.2015.07.124

[CR102] Palladino G, Rampelli S, Scicchitano D, Musella M, Quero GM, Prada F, Mancuso A, Seyfarth AM, Turroni S, Candela M, Biagi E (2021) Impact of marine aquaculture on the microbiome associated with nearby holobionts: the case of *Patella**caerulea* living in proximity of sea bream aquaculture cages. Microorganisms 9(2):455. 10.3390/microorganisms9020455 33671759 10.3390/microorganisms9020455 PMC7927081

[CR103] Panteli N, Mastoraki M, Lazarina M, Chatzifotis S, Mente E, Kormas KAR, Antonopoulou E (2021) Configuration of gut microbiota structure and potential functionality in two teleosts under the influence of dietary insect meals. Microorganisms 9:699. 10.3390/microorganisms9040699 33800578 10.3390/microorganisms9040699 PMC8067204

[CR104] Peters G (2018) Userfriendlyscience: quantitative analysis made accessible. 10.17605/osf.io/txequ

[CR105] Petriglieri F, Kondrotaite Z, Singleton C, Nierychlo M, Dueholm MKD, Nielsen PH (2023) A comprehensive overview of the Chloroflexota community in wastewater treatment plants worldwide. mSystems 8:e00667-23. 10.1128/msystems.00667-23 37992299 10.1128/msystems.00667-23 PMC10746286

[CR106] Pham LN, Kanther M, Semova I, Rawls JF (2008) Methods for generating and colonizing gnotobiotic zebrafish. Nat Protoc 3:1862. 10.1038/nprot.2008.186 19008873 10.1038/nprot.2008.186 PMC2596932

[CR107] Poore J, Nemecek T (2018) Reducing food’s environmental impacts through producers and consumers. Science 360:987–992. 10.1126/science.aaq0216 29853680 10.1126/science.aaq0216

[CR108] Qin C, Zhang Y, Liu W, Xu L, Yang Y, Zhou Z (2014) Effects of chito-oligosaccharides supplementation on growth performance, intestinal cytokine expression, autochthonous gut bacteria and disease resistance in hybrid tilapia *Oreochromis**niloticus* ♀ × *Oreochromis**aureus* ♂. Fish Shell Immunol 40:267–274. 10.1016/j.fsi.2014.07.010 10.1016/j.fsi.2014.07.010 25038280

[CR109] Qin P, Wang T, Luo Y (2022) A review on plant-based protein from soybean: health benefits and soy product development. J Agric Food Res 7:100265. 10.1016/j.jafr.2021.100265

[CR110] Quero GM, Piredda R, Basili M, Maricchiolo G, Mirto S, Manini E, Seyfarth AM, Candela M, Luna GM (2023) Host-associated and environmental microbiomes in an open-sea Mediterranean gilthead sea bream fish farm. Microb Ecol 86(2):1319–1330. 10.1007/s00248-022-02120-7 36205738 10.1007/s00248-022-02120-7 PMC10335962

[CR111] Randazzo B, Zarantoniello M, Gioacchini G, Giorgini E, Truzzi C, Notarstefano V, Cardinaletti G, Huyen KT, Carnevali O, Olivotto I (2020a) Can insect-based diets affect zebrafish (*Danio rerio*) reproduction? A Multidisciplinary Study. Zebrafish 17(5):287–304. 10.1089/zeb.2020.1891 32857683 10.1089/zeb.2020.1891

[CR112] Randazzo B, Zarantoniello M, Gioacchini G, Cardinaletti G, Belloni A, Giorgini E, Faccenda F, Cerri R, Tibaldi E, Olivotto I (2021) Physiological response of rainbow trout (*Oncorhynchus**mykiss*) to graded levels of *Hermetia**illucens* or poultry by-product meals as single or combined substitute ingredients to dietary plant proteins. Aquaculture 538:736550. 10.1016/j.aquaculture.2021.736550

[CR113] Randazzo B, Zarantoniello M, Secci G, Faccenda F, Fava F, Marzorati G, Belloni A, Maradonna F, Orazi V, Cerri R, Povinelli M, Parisi G, Giorgini E, Olivotto I (2022) Towards the identification of a suitable commercial diet for carpione (*Salmo**carpio*, Linnaeus 1758): a multidisciplinary study on fish performances, animal welfare and quality traits. Animals 12:1918. 10.3390/ani12151918 35953906 10.3390/ani12151918 PMC9367350

[CR114] Randazzo B, Di Marco P, Zarantoniello M, Daniso E, Cerri R, Finoia MG, Capoccioni F, Tibaldi E, Olivotto I (2023) Cardinaletti G (2023) Effects of supplementing a plant protein-rich diet with insect, crayfish or microalgae meals on gilthead sea bream (*Sparus**aurata*) and European seabass (*Dicentrarchus**labrax*) growth, physiological status and gut health. Aquaculture 575:739811. 10.1016/j.aquaculture.2023.739811

[CR115] Rangel F, Enes P, Gasco L, Gai F, Hausmann B, Berry D, Oliva-Teles A, Serra CR, Pereira FC (2022) Differential modulation of the European sea bass gut microbiota by distinct insect meals. Front Microbiol 13:831034. 10.3389/fmicb.2022.831034 35495644 10.3389/fmicb.2022.831034 PMC9041418

[CR116] Ratika E, Dharma AP, Annisa R (2020) Combination of *Hermetia**illucens* L maggot flour with fish feed against growth of Sangkuriang catfish (*Clarias* sp.). Syst Rev Pharm 11:529–535. 10.5530/srp.2020.1.66

[CR117] Rimoldi S, Gini E, Iannini F, Gasco L, Terova G (2019) The effects of dietary insect meal from *Hermetia**illucens* prepupae on autochthonous gut microbiota of rainbow trout (*Oncorhynchus**mykiss*). Animals 9(4):143. 10.3390/ani9040143 30987067 10.3390/ani9040143 PMC6523354

[CR118] Rimoldi S, Torrecillas S, Montero D, Gini E, Makol A, Valdenegro VV, Izquierdo M, Terova G (2020) Assessment of dietary supplementation with galactomannan oligosaccharides and phytogenics on gut microbiota of European sea bass (*Dicentrarchus labrax*) fed low fishmeal and fish oil based diet. PloS one. 15(4):e0231494 32298317 10.1371/journal.pone.0231494 PMC7162502

[CR119] Rimoldi S, Antonini M, Gasco L, Moroni F, Terova G (2021) Intestinal microbial communities of rainbow trout (*Oncorhynchus**mykiss*) may be improved by feeding a *Hermetia**illucens* meal/low-fishmeal diet. Fish Physiol Biochem 47:365–380. 10.1007/s10695-020-00918-1 33389354 10.1007/s10695-020-00918-1 PMC8026480

[CR120] Rimoldi S, Ceccotti C, Brambilla F, Faccenda F, Antonini M, Terova G (2023) Potential of shrimp waste meal and insect exuviae as sustainable sources of chitin for fish feeds. Aquaculture 567:739256. 10.1016/j.aquaculture.2023.739256

[CR121] Rimoldi S, Di Rosa AR, Oteri M, Chiofalo B, Hasan I, Saroglia M, Terova G (2024) The impact of diets containing *Hermetia illucens* meal on the growth, intestinal health, and microbiota of gilthead seabream (*Sparus aurata*). Fish Physiol Biochem 50(3):1003–1024. 10.1007/s10695-024-01314-9 38386264 10.1007/s10695-024-01314-9 PMC11213805

[CR122] Rix RR, Guedes RNC, Cutler GC (2022) Hormesis dose–response contaminant-induced hormesis in animals. Curr Opin Toxicol 30:100336. 10.1016/j.cotox.2022.02.009

[CR123] RStudio Team (2020) RStudio: integrated development for R. URLRStudio, PBC, Boston, MA. http://www.rstudio.com/

[CR124] Ruiz A, Andree KB, Furones D, Holhorea PG, Calduch-Giner JÀ, Viñas M, Pérez-Sànchez J, Gisbert E (2023a) Modulation of gut microbiota and intestinal immune response in gilthead seabream (*Sparus**aurata*) by dietary bile salt supplementation. Front Microbiol 14:1123716. 10.3389/fmicb.2023.1123716 37168118 10.3389/fmicb.2023.1123716 PMC10166234

[CR125] Ruiz A, Sanahuja I, Andree KB, Furones D, Holhorea PG, Calduch-Giner JA, Pastor JJ, Viñas M, Pérez-Sánchez J, Morais S, Gisbert E (2023) The potential of a combination of pungent spices as a novel supplement in gilthead seabream (*Sparus aurata*) diets to aid in the strategic use of fish oil in aquafeeds: a holistic perspective. Front Immunol 14:1222173. 10.3389/fimmu.2023.1222173 37818366 10.3389/fimmu.2023.1222173 PMC10561386

[CR126] Skrivanova E, Marounek M, Benda V, Brezina P (2006) Susceptibility of Escherichia coli, Salmonella sp. and Clostridium perfringens to organic acids and monolaurin. Vet-Med Czech 51(3):81–88. 10.17221/5524-vetmed

[CR127] Spranghers T, Michiels J, Vrancx J, Ovyn A, Eeckhout M, De Clercq P, De Smet S (2018) Gut antimicrobial effects and nutritional value of black soldier fly (*Hermetia illucens* L.) prepupae for weaned piglets. Anim Feed Sci Technol 235:33–42. 10.1016/j.anifeedsci.2017.08.012 10.1002/jsfa.8081 27734508

[CR128] Stenberg OK, Holen E, Piemontese L, Liland NS, Lock EJ, Espe M, Belghit I (2019) Effect of dietary replacement of fish meal with insect meal on in vitro bacterial and viral induced gene response in Atlantic salmon (*Salmo**salar*) head kidney leukocytes. Fish Shellfish Immunol 91:223–232. 10.1016/j.fsi.2019.05.042 31121289 10.1016/j.fsi.2019.05.042

[CR129] Sutcliffe IC (2010) A phylum level perspective on bacterial cell envelope architecture. Trends Microbiol 18(10):464–470. 10.1016/j.tim.2010.06.005 20637628 10.1016/j.tim.2010.06.005

[CR130] Tacon A, Metian M (2015) Feed matters: satisfying the feed demand of aquaculture. Rev Fish Sci Aquac 23:1–10. 10.1080/23308249.2014.987209

[CR131] Tacon AGJ, Metian M, McNevin AA (2022) Future feeds: suggested guidelines for sustainable development. Rev Fish Sci Aquac 30:135–142. 10.1080/23308249.2020.1860474

[CR132] Tarnecki AM, Burgos FA, Ray CL, Arias CR (2017) Fish intestinal microbiome: diversity and symbiosis unravelled by metagenomics. J Appl Microbiol 123(1):2–17. 10.1111/jam.13415 28176435 10.1111/jam.13415

[CR133] Terova G, Rimoldi S, Ascione C, Gini E, Ceccotti C, Gasco L (2019) Rainbow trout (*Oncorhynchus**mykiss*) gut microbiota is modulated by insect meal from *Hermetia**illucens* prepupae in the diet. Rev Fish Biol Fish 29(2):465–486. 10.1007/s11160-019-09558-y

[CR134] Terova G, Gini E, Gasco L, Moroni F, Antonini M, Rimoldi S (2021) Effects of full replacement of dietary fishmeal with insect meal from *Tenebrio**molitor* on rainbow trout gut and skin microbiota. J Animal Sci Biotechnol 12:30. 10.1186/s40104-021-00551-9 10.1186/s40104-021-00551-9 PMC7860006 33536078

[CR135] Tippayadara N, Dawood MA, Krutmuang P, Hoseinifar SH, Doan HV, Paolucci M (2021) Replacement of fish meal by black soldier fly (*Hermetia illucens*) larvae meal: effects on growth, haematology, and skin mucus immunity of Nile Tilapia Oreochromis niloticus. Animals 11(1):193. 10.3390/ani11010193 33467482 10.3390/ani11010193 PMC7830215

[CR136] Tocher DR (2010) Fatty acid requirements in ontogeny of marine and freshwater fish. Aquac Res 41:717–732. 10.1111/j.1365-2109.2008.02150.x

[CR137] Turkmen S, Hernández-Cruz CM, Zamorano MJ, Fernández-Palacios H, Montero D, Afonso JM, Izquierdo M (2019) Long-chain PUFA profiles in parental diets induce long-term effects on growth, fatty acid profiles, expression of fatty acid desaturase 2 and selected immune system-related genes in the offspring of gilthead seabream. Br J Nutr 122:25–38. 10.1017/S0007114519000977 31266551 10.1017/S0007114519000977

[CR138] UN DESA (2023) The sustainable development goals report 2023: special edition - July 2023; UN DESA: New York, NY, USA, 2023

[CR139] Vagner M, Santigosa E (2011) Characterization and modulation of gene expression and enzymatic activity of delta-6 desaturase in teleosts: a review. Aquaculture 315:131–143. 10.1016/j.aquaculture.2010.11.031

[CR140] van Zanten HHE, Oonincx DGAB, Mollenhorst H, Bikker B, Meerburg BG, de Boer IGM (2014) Can the environmental impact of livestock feed be reduced by using waste-fed housefly larvae? In: Proceedings of the 9th International Life Cycle Assessment of Foods Conference (LCA Food 2014) 1455–1461. https://edepot.wur.nl/331305. Accessed 5 Sept 2024

[CR141] Vargas-Abúndez AJ, Randazzo B, Foddai M, Sanchini L, Truzzi C, Giorgini E, Gasco L, Olivotto I (2019) Insect meal based diets for clownfish: biometric, histological, spectroscopic, biochemical and molecular implications. Aquaculture 498:1–11. 10.1016/j.aquaculture.2018.08.018

[CR142] Watanabe T, Pongmaneerat J, Sato S, Takeuchi T (1993) Replacement of fish meal by alternative protein sources in rainbow trout diets. Nippon Suisan Gakk 59(9):1573–1579. 10.2331/suisan.59.1573

[CR143] Weththasinghe P, Rocha SDC, Øyås O, Lagos L, Hansen JØ, Mydland LT, Øverland M (2022) Modulation of Atlantic salmon (*Salmo**salar*) gut microbiota composition and predicted metabolic capacity by feeding diets with processed black soldier fly (*Hermetia**illucens*) larvae meals and fractions. Anim Microbiome 4:9. 10.1186/S42523-021-00161-w 35033208 10.1186/s42523-021-00161-w PMC8760679

[CR144] Wickham H (2016) Programming with ggplot2. In ggplot2 2016 (pp. 241–253). Springer, Cham. 10.1007/978-3-319-24277-4_12

[CR145] Xia Y, Lu M, Chen G, Cao J, Gao F, Wang M, Liu Z, Zhang D, Zhu H, Yi M (2018) Effects of dietary *Lactobacillus rhamnosus* JCM1136 and *Lactococcus lactis* subsp. lactis JCM5805 on the growth, intestinal microbiota, morphology, immune response and disease resistance of juvenile Nile tilapia Oreochromis Niloticus. Fish Shellfish Immunol 76:368–379. 10.1016/j.fsi.2018.03.020 29550602 10.1016/j.fsi.2018.03.020

[CR146] Xu X, Ji H, Yu H, Zhou J (2020) Influence of dietary black soldier fly (*Hermetia**illucens* Linnaeus) pulp on growth performance, antioxidant capacity and intestinal health of juvenile mirror carp (*Cyprinus**carpio* var. specularis). Aquac Nutr 26:432–443. 10.1111/anu.13005

[CR147] Yang C, Mai J, Cao X, Burberry A, Cominelli F, Zhang L (2023) ggpicrust2: an R package for PICRUSt2 predicted functional profile analysis and visualization. Bioinformatics 39(8):btad470. 10.1093/bioinformatics/btad470 37527009 10.1093/bioinformatics/btad470 PMC10425198

[CR148] Yi Y, Wang H, Chen Y, Gou M, Xia ZY, Hu B, Nie Y, Tang YQ (2020) Identification of novel butyrate- and acetate-oxidizing bacteria in butyrate-fed mesophilic anaerobic chemostats by DNA-based stable isotope probing. Microb Ecol 79:285–298. 10.1007/s00248-019-01400-z 31263981 10.1007/s00248-019-01400-z

[CR149] Yokokawa T, Nagata T (2010) Linking bacterial community structures to carbon fluxes in marine environments. J Oceanogr 66:1–12. 10.1007/s10872-010-0001-4

[CR150] Zarantoniello M, Bruni L, Randazzo B, Vargas A, Gioacchini G, Truzzi C, Annibaldi A, Riolo P, Parisi G, Cardinaletti G, Tulli F, Olivotto I (2018) Partial dietary inclusion of *Hermetia**illucens* (black soldier fly) full-fat prepupae in zebrafish feed: biometric, histological, biochemical, and molecular implications. Zebrafish 15:519–532. 10.1089/zeb.2018.1596 29912648 10.1089/zeb.2018.1596

[CR151] Zarantoniello M, Randazzo B, Nozzi V, Truzzi C, Giorgini E, Cardinaletti G, Freddi L, Ratti S, Girolametti F, Osimani A, Notarstefano V (2021) Physiological responses of Siberian sturgeon (*Acipenser baerii*) juveniles fed on full-fat insect-based diet in an aquaponic system. Sci Rep 11(1):1057. 10.1038/s41598-020-80379-x 33441819 10.1038/s41598-020-80379-x PMC7806854

[CR152] Zarantoniello M, Pulido Rodriguez LF, Randazzo B, Cardinaletti G, Giorgini E, Belloni A, Secci G, Faccenda F, Pulcini D, Parisi G, Capoccioni F, Tibaldi E, Olivotto I (2022a) Conventional feed additives or red claw crayfish meal and dried microbial biomass as feed supplement in fish meal-free diets for rainbow trout (*Oncorhynchus mykiss*): possible ameliorative effects on growth and gut health status. Aquaculture 554:738137. 10.1016/j.aquaculture.2022.738137

[CR153] Zarantoniello M, Randazzo B, Secci G, Notarstefano V, Giorgini E, Lock EJ, Parisi G, Olivotto I (2022b) Application of laboratory methods for understanding fish responses to black soldier fly (*Hermetia illucens*) based diets. J Insects Food Feed 8(11):1173–1195. 10.3920/JIFF2020.0135

[CR154] Zhang C, Li Y, Yu H, Li T, Ye L, Zhang X, Wang C, Li P, Ji H, Gao Q, Dong S (2024) Co-exposure of nanoplastics and arsenic causes neurotoxicity in zebrafish (*Danio rerio*) through disrupting homeostasis of microbiota-intestine-brain axis. Sci Total Environ 912:169430. 10.1016/j.scitotenv.2023.169430 38135083 10.1016/j.scitotenv.2023.169430

