# Supplementary material for: Effect of graded inclusion of black soldier fly (Hermetia illucens, Linnaeus, 1758) pre-pupae meal in diets for gilthead seabream (Sparus aurata, Linnaeus, 1758) on gut microbiome and liver morphology
Source: Fish Physiol Biochem. 2025 Apr 22;51(3):85. doi: 10.1007/s10695-025-01485-z (PMC12014712; doi:10.1007/s10695-025-01485-z)
Supplement: Supplementary file 1 — Supplementary file1 (DOCX 302 KB) [file 10695_2025_1485_MOESM1_ESM.docx]

**Supplementary material**

**EFFECT OF GRADED INCLUSION OF BLACK SOLDIER FLY (*Hermetia illucens*, Linnaeus, 1758) PRE-PUPAE MEAL IN DIETS FOR GILTHEAD SEABREAM (*Sparus aurata*, Linnaeus, 1758) ON GUT MICROBIOME AND LIVER MORPHOLOGY.**

Marco Basili^1,2✧^, Basilio Randazzo^3,4✧^, Letteria Caccamo^3,4^, Stefano Guicciardi^2^, Martina Meola^3^, Anna Perdichizzi^3,4^, Grazia Marina Quero^2,4*^, Giulia Maricchiolo^3,4^

*^1^Alma Mater Studiorum—University of Bologna, Bologna, Italy*

*^2^IRBIM-Institute of Marine Biological Resources and Biotechnologies, CNR-National Research Council, Largo Fiera della Pesca 1, 60125 Ancona, AN, Italy*

^3^*IRBIM-Institute of Marine Biological Resources and Biotechnologies, CNR-National Research Council, Spianata S. Raineri, 98122 Messina, Italy*

^4^*National Biodiversity Future Center (NBFC), Piazza Marina 61, 90133 Palermo, Italy*

^✧^*These Authors contributed equally to this work*

^*^*Corresponding Author*

Marco Basili: [marco.basili@irbim.cnr.it](mailto:marco.basili@irbim.cnr.it)

<https://orcid.org/0000-0002-9936-9507>

Basilio Randazzo: [basilio.randazzo@cnr.it](mailto:basilio.randazzo@cnr.it)

<https://orcid.org/0000-0002-5032-413X>

Letteria Caccamo: [letteria.caccamo@cnr.it](mailto:letteria.caccamo@cnr.it)

<https://orcid.org/0000-0001-8599-163X>

Stefano Guicciardi: [stefano.guicciardioguizzardi@cnr.it](mailto:stefano.guicciardioguizzardi@cnr.it)

<https://orcid.org/0000-0003-3539-2828>

Martina Meola: [martina.meola@irbim.cnr.it](mailto:martina.meola@irbim.cnr.it)

<https://orcid.org/0009-0000-4830-5020>

Anna Perdichizzi: [anna.perdichizzi@cnr.it](mailto:anna.perdichizzi@cnr.it)

<https://orcid.org/0000-0002-8285-8013>

Grazia Marina Quero: [graziamarina.quero@cnr.it](mailto:graziamarina.quero@cnr.it)

<https://orcid.org/0000-0002-2562-1255>

Giulia Maricchiolo: [giulia.maricchiolo@cnr.it](mailto:giulia.maricchiolo@cnr.it)

<https://orcid.org/0000-0002-5670-6243>


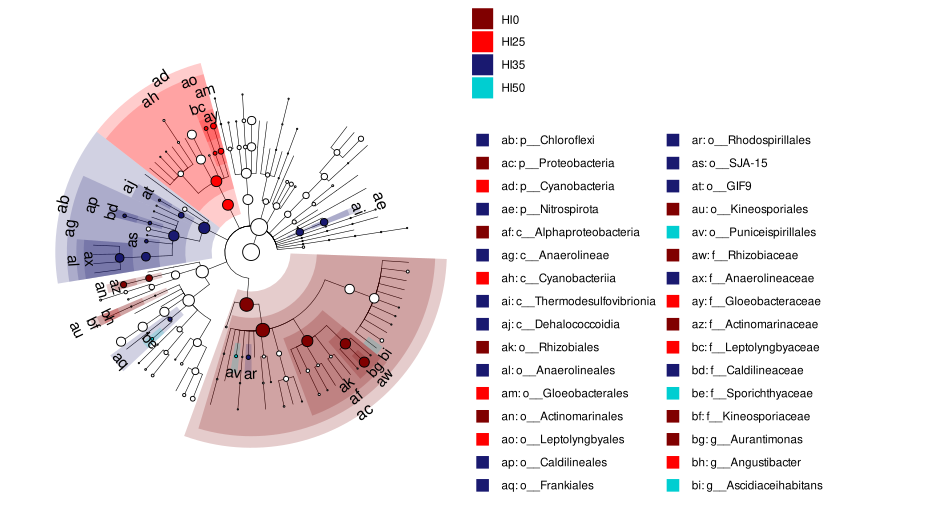


**Supplementary Figure 1.** Taxonomic cladogram using linear discriminant analysis effect size (LEfSe) comparing microbiomes grouped by Diet.


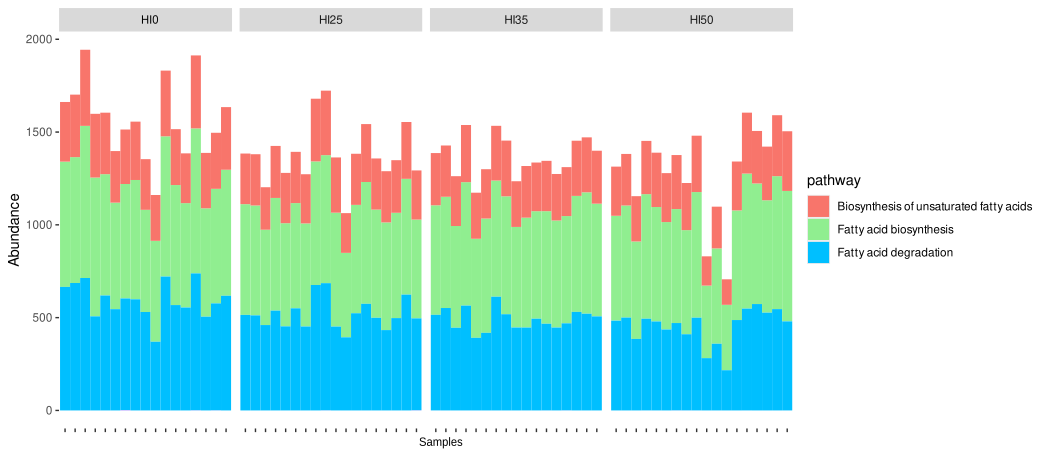


**Supplementary Figure 2.** Abundance of fatty acid gene families from Picrust results across the different diets.
